# Pest consumption by common bats in diverse landscapes

**DOI:** 10.1371/journal.pone.0347853

**Published:** 2026-06-17

**Authors:** Miren Aldasoro, Oihane Diaz de Cerio, Nerea Vallejo, Lander Olasagasti, Joxerra Aihartza

**Affiliations:** Department of Zoology and Animal Cell Biology, Faculty of Science and Technology, University of the Basque Country EHU, Leioa, The Basque Country; University of Illinois at Urbana-Champaign, UNITED STATES OF AMERICA

## Abstract

Insectivorous bats are increasingly recognised for their role in suppressing agricultural pests. To better understand the value of their ecosystem services, it is pertinent to assess their predation quantitatively. In this context, studying frequent anthropophilic bats that form large colonies is particularly interesting, as the ecosystem services they provide are more substantial. Building on this motivation, we analysed the diet of six horseshoe bat colonies—three *Rhinolophus hipposideros* and three *R. ferrumequinum*—across different landscapes within their optimal distribution area, focusing on their consumption of those pests. We found that these bats consistently consumed a variety of pests, with consumption correlating with outbreaks of the pest species. Some of these species were previously unknown in the area or were not fought against by farmers. This underscores the value of bats as sensitive trackers of current and emerging insect communities and potential pests, as they can detect and consume species that may go unnoticed or unmonitored by conventional human surveys. Additionally, we estimated the overall pest consumption by these bat species in the study area and assessed consumption at the colony level. Our estimates indicate that pest consumption during the breeding season totalled nearly 3 tons of insects. These findings underscore the importance of maintaining favourable habitats for common bats as they can play a vital role in controlling and mitigating ecosystem imbalances caused by changes in farming practices, landscape disturbances, or climate change.

## Introduction

Insectivorous bats feed on a wide variety of arthropods, including many pest species such as, moths, beetles, and other insects that can damage crops and forests (e.g., [1–4]). By acting as natural consumers of these insects, bats function as effective suppressors of pest populations within the ecosystems they inhabit [5–8]. Thus, bats can offer essential ecosystem services that help mitigate environmental and economic impacts of pest outbreaks, reducing the need for chemical pest control [6,9].

Seasonal dietary studies have revealed the remarkable adaptability of bats in their feeding habits. They exhibit high dietary plasticity, shifting their prey use in response to changes in local insect availability, including sudden pest outbreaks [10–15]. Bats often adjust their foraging areas to those with greater prey availability [16], forage opportunistically [14], and even consistently consume insects originating in habitats outside their typical hunting grounds [17]. Because bats adjust their diets and foraging habitats, they are highly effective pest suppressors, as many agricultural pest insects tend to have cyclic outbreaks [18].

Faecal DNA metabarcoding has become a powerful tool for characterizing bat diets, allowing researchers to detect a wide range of prey, including numerous agricultural and forestry pests [4,19]. Despite technical challenges in obtaining reliable quantitative estimates—stemming from of faecal DNA (e.g., [20–22]) and can be difficult even with targeted qPCR [10], metabarcoding still provides detailed insight into which pest species bats consume. Moreover, studies that focus specifically on pest suppression have estimated consumption rates using qPCR or metabarcoding [10,14]. Exclosure experiments consistently demonstrate that bats play a key role in reducing crop-damaging insect populations (e.g., [23–27]). While estimates of pest consumption by bats are approximations, given variation in their feeding patterns and dependence on prey availability across landscapes, it is crucial to understand the extent of bat pest consumption across various agroecosystems. This understanding is key to discussing pest regulation and evaluating the actual value of bat insectivory as an ecosystem service [28].

Abundant bat species are more likely to act as significant pest suppressors, as effective predation requires a high density of predators (e.g., [7,9,29,30]). They can form large, stable colonies, amplifying their impact on pest suppression. Since effective predation (potentially leading to control of pest populations) requires a high predator density [28], larger populations can collectively consume more pests. The larger the population, the greater the collective consumption of pests, leading to a more significant reduction in harmful insect populations. This collective impact underscores the significance of bat populations in pest suppression, reinforcing the need to protect both these species and their habitats to maximise their ecological contributions [28].

The Greater Horseshoe bat *Rhinolophus ferrumequinum* (Schreber 1774) and the Lesser Horseshoe bat *Rhinolophus hipposideros* (Bechstein 1800) are widely distributed cave-dwelling species in karstic areas where roosting is not a limiting factor*. R. ferrumequinum* is a larger species and emits higher frequency calls than *R. hipposideros* [31,32]. These species are abundant in regions such as the Northern Iberian Peninsula [33,34], forming maternity colonies of several hundred individuals. Our study examines which pest species are most frequently consumed by these two widespread bat species across their active season and across different agroforestry landscapes. By identifying the key pests in their diet and estimating the total biomass consumed, we aim to clarify the extent to which these bats contribute to natural pest suppression. Together, these insights help highlight the ecological and agricultural importance of their foraging activity.

## Materials and methods

### Ethics statement

This study did not involve animal handling, as the samples were collected non-invasively using paper collectors placed beneath the colony, thereby avoiding animal disturbances. Therefore, the ethics committee of the University of the Basque Country (EHU) has granted this study an ethical exemption because it is not considered a “procedure” under the Spanish Royal Decree 53/2013, which establishes the basic standards applicable to the protection of animals used in experiments and other scientific purposes.

### Study area and colonies

**Fig 1:**
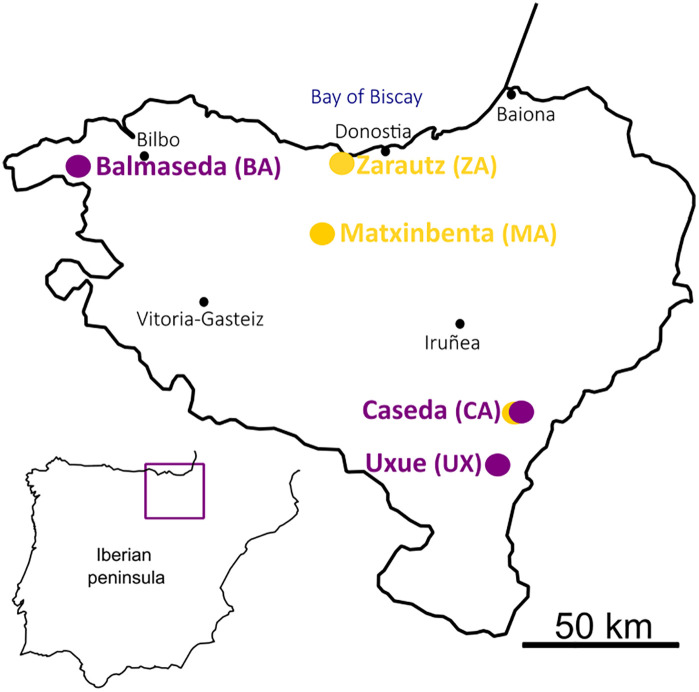
Geographic distribution of sampled bat colonies in the Basque Country. The main map illustrates the five primary sampling locations across the northern Iberian Peninsula. Points are colour-coded by species: purple represents *Rhinolophus ferrumequinum* (Rfe) and yellow represents *R. hipposideros* (Rhi). The split-colored marker at Caseda (CA) indicates a site where both species were sampled. The inset (bottom left) depicts the location of the study area within the Iberian Peninsula and its position relative to the Spanish-French border.

The study was carried out in the Basque Country, northern Iberian Peninsula. Three *Rhinolophus ferrumequinum* and three *R. hipposideros* roosts were sampled (Fig 1), with significant differences in land use and climate (Table 1).

**Table 1:** Characteristics of the six sampled colonies, including species, population size (pre-birth), climate classification (Köppen-Geiger system),associated main crops and habitats, cropland-orchard and urban percentages in a 3 km buffer. Rfe: *R. ferrumequinum*; Rhi: *R. hipposideros*. Köppen-Geiger climate classification: Cfb: West coast maritime climate; Csb: Mediterranean temperate climate; Csa: Mediterranean climate. [35].

Colony name	Species	Size	Climate	Main crops and habitats	Crop-orchard %	Urban %
Balmaseda (BA)	Rfe	300	Cfb	Pine plantations, pastures	0.75	8.46
Caseda (CA Rf)	Rfe	150	Csb	Cereals, vineyards and orchards, Mediterranean bush, pine plantation	37.25	5.33
Uxue (UX)	Rfe	150	Csa	Orchards, Mediterranean shrubland, forest.	24.61	2.09
Zarautz (ZA)	Rhi	150	Cfb	Vineyards, fruit trees, pastures, deciduous and coniferous forests	8.66	32.56
Matxinbenta (MA)	Rhi	200	Cfb	Deciduous and conifer forests, meadows, and pasture	0.24	1.64
Caseda (CA Rh)	Rhi	30	Csb	Cereals, vineyards and orchards, Mediterranean bush, pine plantation	37.47	4.94

### Sampling bat droppings

In 2022, we sampled colonies of both *Rhinolophus ferrumequinum* and *Rhinolophus hipposideros* throughout the maternity season, using non-invasive methods to avoid disturbing the animals. Sampling for *R. ferrumequinum* began in Julian Week 9 at UX (March), with BA and CArf colonies following in Week 15 (mid April), while *R. hipposideros* sampling started in Julian Week 17 (late April) across the MA, ZA, and CA-Rh colonies. UX bats left first on Week 27 (beginning of July), followed by CA-Rf bats in Week 35 (late August) and BA bats in Week 37 (mid-September), while MA bats stayed until Week 39 (end of September), two weeks longer than the ZA and CA-Rh colonies (Week 37). Paper sheets were placed under the bats every fortnight to collect the freshest faeces, preserving the DNA quality.

We collected up to 24 small “community samples” of four to six pellets each for each sampling event (adapted from [36,37]). These samples aim to reflect the diet of the entire maternity colony throughout the sampling period, rather than the contributions from individual animals. We aimed to collect the freshest, most intact pellets to ensure the best quality DNA. In some colonies of *R. ferrumequinum,* mixed with *Myotis emarginatus*, we selected the larger pellets because they most likely belonged to *R. ferrumequinum*. All in all, 413 community samples were collectedfor *R. hipposideros* and 377 for *R. ferrumequinum* for sequencing and analysis.

In some *R. ferrumequinum* colonies that were mixed with *Myotis emarginatus*, sequencing revealed DNA contamination in a subset of samples. We therefore removed the contaminated samples and merged the remaining clean ones, analysing them at the monthly rather than weekly level to maintain a statistically robust sample size. This procedure yielded 237 clean samples. After filtering the samples, we examined any possible correlation between *Myotis emarginatus* Operational Taxonomic Units (OTUs) and prey species. No clear correlations were found, so we assumed that the analysed species in the diet belonged to the prey of *Rhinolophus ferrumequinum*. In the case of *R. hipposideros*, no contamination issues were detected, so the sample size remained sufficient to retain the weekly analysis.

### DNA extraction, PCR amplification, library preparation and sequencing

We extracted DNA from frozen individual faecal samples (up to 280 mg) using the Kingfisher extraction tool (Thermo Fisher Scientific Inc.) after homogenising them with the Precellys 24 Touch Homogeniser (Bertin Technologies SAS, France). From the extracts, two 178- and 180-bp different mini-COI segments of the mitochondrial DNA cytochrome c oxidase subunit I (COI) barcode region were amplified with FWH1 (fwhF1/fwhF2) [38] and ANML (CO1490/CO1‐CFMRa) [39] primers, respectively. Amplifications were performed using the QIAGEN Multiplex PCR Kit (Qiagen Iberia, S.L. Madrid) in 25 μl PCR reactions, according to the manufacturer’s protocols. Libraries were built using Illumina’s Nextera XT kit, and samples were sequenced on the Illumina NovaSeq platform (SP Flow Cell kit v2 PE: 2 x 250 bp (500 cycles)/ 800Mreads). PCR amplification, DNA library construction and sequencing processes were carried out at the Genomics and Proteomics General Service (SGIker) of the University of the Basque Country.

### Bioinformatic analyses

We performed primer-based separation, quality control, sequence pre-processing, and collapsed identical sequences into single sequences using CUTADAPT [40] and VSEARCH [41]. We clustered sequences into Operational Taxonomic Units (OTU) by VSEARCH at a 97% similarity threshold [42] using the “–cluster_size” command. Subsequently, we cleaned up chimaera OTUs with USEARCH’s “-uchime3_denovo” command. Only OTUs with an abundance higher than 0.5% in any sample unit were selected for the analysis.

The taxonomic assignment of each OTU was performed by comparing the representative sequence against reference sequences in GenBank (www.ncbi.nlm.nih.gov; date 2023-08-28) using the “-blastn” command [43]. Only hits with pairwise identity above 98% and e-values below 1e-20 were accepted [44,45] to ensure that the match did not occur by chance. We downloaded the arthropod species databases for Spain, France and Portugal from GBIF (www.gbif.org; date 2022-11-03) to verify that the identified species encompassed our study area. OTUs with multiple potential assignments were carefully reviewed and manually refined to assign the most accurate taxa to each OTU based on pairwise identity and species distribution.

### Diet analysis and pest assessment

We only used OTUs classified as potential prey for the analysis, with data converted to weighted percentages of occurrences (wPOO)—a metric based on presence/absence data that provides a good proxy of consumption [22,46]— and frequency of occurrence (FOO) [22]. Full diet details can be found in [47,48]. We compared the species identified in the diet with pest databases, mainly the Integrated Pest Management Guides of the Spanish Government [49]. Core diets were analysed by selecting the species with a Frequency of Occurrence (FOO) above 5% in each colony [50].

### A quantitative approach to pest insect consumption

Acknowledging the methodological uncertainties mentioned in the introduction, these estimations aim to assess a range of pest consumption. As an initial quantitative approach, we estimated pest consumption for each colony over the full five-month sampling season. We first estimated the total pest consumption ranges for each species in the Southern (Iberian part) Basque Country (17,625 km²) and then calculated the consumption rates for each colony based on their population sizes and mean pest consumption values (Supporting Information 1), using the following formula:

**Pest Consumption (g)** = *c* x *mean weight* x *pop size* x *wPOO*

***c***: Night prey consumption estimates. c_1:_ Kurta et al. [51] estimated that in the temperate insectivorous bat *Myotis lucifugus*, individuals should daily eat about 70% of their body weight in insects to fulfil their requirements during pregnancy and lactation; c_2_: different studies estimated this value on the 30% in diverse species (e.g., [52–55]). We used 30% and 70% as approximate lower and upper bounds to capture the variability reported in the literature across species, seasons, and reproductive stages.

***mean weight***: Species mean weight (23.5 g for *R. ferrumequinum* and 6.5 g for *R. hipposideros*, based on the information found in the Handbook of the Mammals of Europe-Chiroptera [31,32].

***pop size***: Population estimates were derived from the research developed in part of the Basque Country by [56], supplemented and validated with our research group’s most recent unpublished data. We extrapolated the available data for the species *R. ferrumequinum* to our study area. For *R. hipposideros*, no information was available; however, since other studies have reported similar population sizes for *R. ferrumequinum* and *R. hipposideros* [33,34], we used the same estimate for both species: 15,000 individuals.

***wPOO****:* Mean pest consumption (wPOO from our study) (Table 2).

**Table 2:** Mean pest consumption of the studied species, overall and for each colony. Species: *R. ferrumequinum: Rhinolophus ferrumequinum; R. hipposideros: Rhinolophus hipposideros*. Sites: BA: Balmaseda; CA Rf: Caseda (*Rhinolophus ferrumequinum*); UX: Uxue; ZA: Zarautz; MA: MAtxinbenta: CA Rh: Caseda (*Rhinolophus hipposideros*).

	Mean pest consumption (wPOO)
***R. ferrumequinum***	**7.65 ± 3.31**
**BA**	10.71 ± 2.66
**CA Rf**	6.54 ± 2.47
**UX**	5.29 ± 2.10
***R. hipposideros***	**8.55 ± 3.97**
**ZA**	11.90 ± 3.65
**MA**	7.88 ± 2.79
**CA Rh**	5.91 ± 3.09

## Results

### R. hipposideros

**Fig 2:**
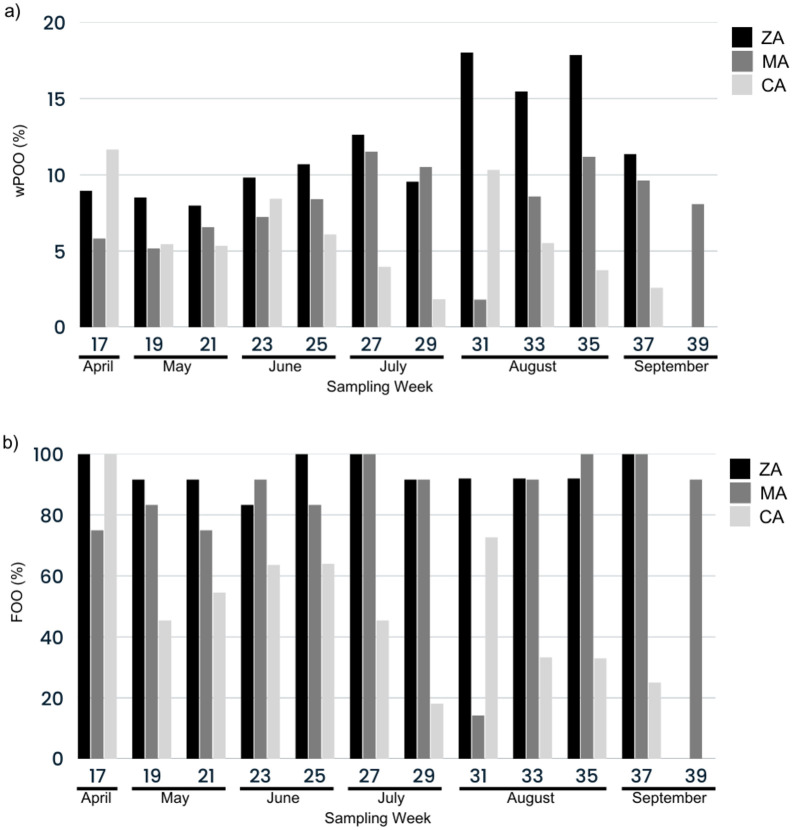
Weekly pest consumption by colonies in (a) wPOO (weighted percentage of occurrence) and (b) FOO (frequency of occurrence). Note that week 39 only has data for the MA colony. ZA: Zarautz; MA: Matxinbenta; CA: Caseda.

We found pests in 304 out of 406 faecal samples. 93.94% of samples were positive for pests in ZA, 79.08% in MA, and 48.76% in CA. In total, 66 identified taxa belong to pest species or genera (Supporting Information 1). We found 40 pest species in the diets of ZA, 45 in MA and 37 in CA. Pest species were consumed throughout the entire season in every colony. The peak of pest consumption in ZA was in August (weeks 31–35); in MA, it was in July (weeks 27 and 29) and at the end of August (week 35); finally, in CA, it was at the end of April (week 17) and the beginning of August (week 31) (Fig 2).

We identified 13 pest species in the core diets of the colonies, primarily associated with orchards and tree plantations (see Table 3). In ZA, where vineyards and fruit trees are economically significant, the pest species were directly linked to these agricultural sectors and horticultural plants (e.g., *Cydia sp., Zeuzera pyrina, Noctua pronuba, N. comes, Autographa gamma*). Similarly, in MA, most pest species were associated with fruit and nut trees. In CA, however, the pest species in the core diet were associated with forest trees (*Cydia fagiglandana* and *Rhyacionia buoliana*).

**Table 3:** Pest species identified on the core diets (FOO > 5%) of *R. hipposideros*, their host species and their abundance in diet (wPOO). Values for individual colonies (MA: Matxinbenta; ZA: Zarautz; CA: Caseda) reflect the proportion within each colony’s samples, whereas the total (TOT) represents the proportion across all samples combined, which may result in lower overall percentages. (TOT: Whole diet; MA: Matxinbenta colony; ZA: Zarautz colony; CA: Caseda colony).

Pest Species	Pest of	TOT	MA	ZA	CA
***Agrotis segetum***	*Beta vulgaris; Brassica oleracea; Capsicum sp.; Lactuca sp.; Medicago sativa; Pisum sativum; Solanum sp.; Vitis vinifera; Zea mays…*	0.52	0.97	0.47	0.00
***Autographa gamma***	*Beta vulgaris; Brassica oleracea; Medicago sativa; Lactuca sp.; Vitis vinifera*	0.62	1.16	0.57	0.00
***Cydia sp.***	Fruit-trees*; Castanea sativa; Juglans regia; Quercus sp.*	0.92	1.39	0.98	0.26
***Cydia fagiglandana***	*Castanea sativa; Quercus sp.*	0.65	0.70	0.40	0.88
***Dioryctria mendacella***	Conifers	0.28	0.35	0.39	0.07
***Drosophila sp.***	Fruit-trees*; Vitis vinifera*	0.41	1.08	0.00	0.00
***Eurydema ornata***	*Brassica oleracea*	0.27	0.44	0.00	0.34
***Noctua comes***	*Vitis vinifera*	0.38	0.18	0.68	0.29
***Noctua pronuba***	*Vitis vinifera*	0.47	0.71	0.49	0.14
***Opogona sacchari***	*Ficus sp.; Solanum tuberosum; Zea mays…*	4.15	0.71	11.90	0.00
***Plutella xylostella***	*Brassica oleracea*	0.31	0.37	0.41	012
***Rhyacionia buoliana***	Conifers	0.31	0.03	0.05	0.95
***Zeuzera pyrina***	*Corylus avellana;* Fruit-trees*; Juglans regia; Olea europaea*	0.44	0.61	0.58	0.08

**Fig 3:**
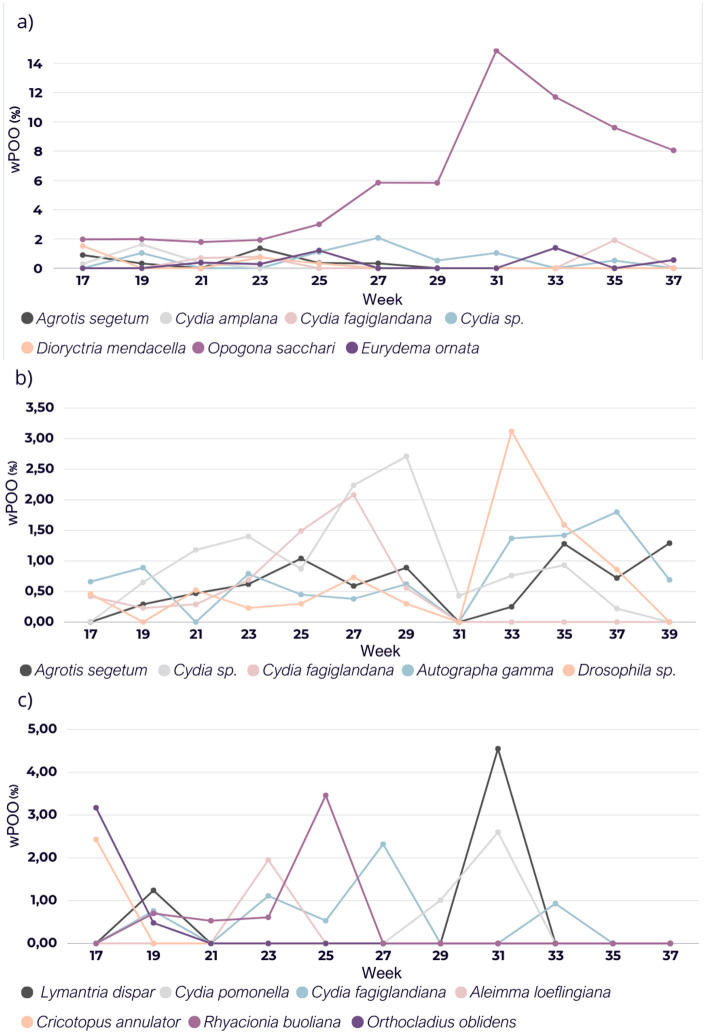
Consumption of pest species during the active season by *Rhinolophus hipposideros.* The species have been selected from the five most consumed per week and colony. Consumption has been calculated as wPOO. **(a)** ZA: Zarautz colony, **(b)** MA: Matxinbenta and **(c)** CA: Caseda.

Additionally, some pest species not part of the colonies’ core diet ranked among the top ten most consumed per week, indicating seasonal variations in consumption (Fig 3). For instance, *Cydia amplana* in ZA has been identified as a pest of fruit trees. In CA, we found five additional pest insect species: one associated with fruit trees and nut trees (*Cydia pomonella),* one with *Quercus sp.* (*Aleimma loeflingiana*) and two with *Oryza sativa* (*Cricotopus annulator* and *Orthocladius oblidens*).

**Fig 4:**
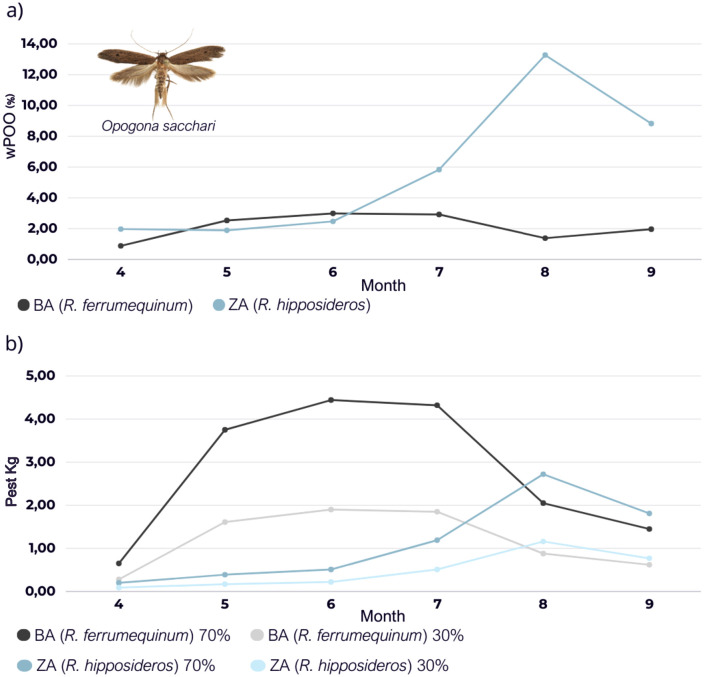
Overall consumption along the season of *Opogona sacchari* in colonies BA (Balmaseda, *R. ferrumequinum*) and ZA (Zarautz, *R. hipposideros*). The horizontal axis shows sampling weeks, and the vertical axis in (a) wPOO and in (b) monthly consumption estimate in kg, calculated from c1 (in dark, following Kurta et al. [51]) and c2 (in light, following several authors, as noted in the text).

Furthermore, we must point out that *Opogona sacchari* was consistently consumed throughout the season (FOO 72.73%) in ZA at a much higher consumption rate than other species. In fact, during week 31, it reached a consumption of 15% wPOO, while the other species did not exceed 5% (Fig 3; Fig 4).

### R. ferrumequinum

**Fig 5:**
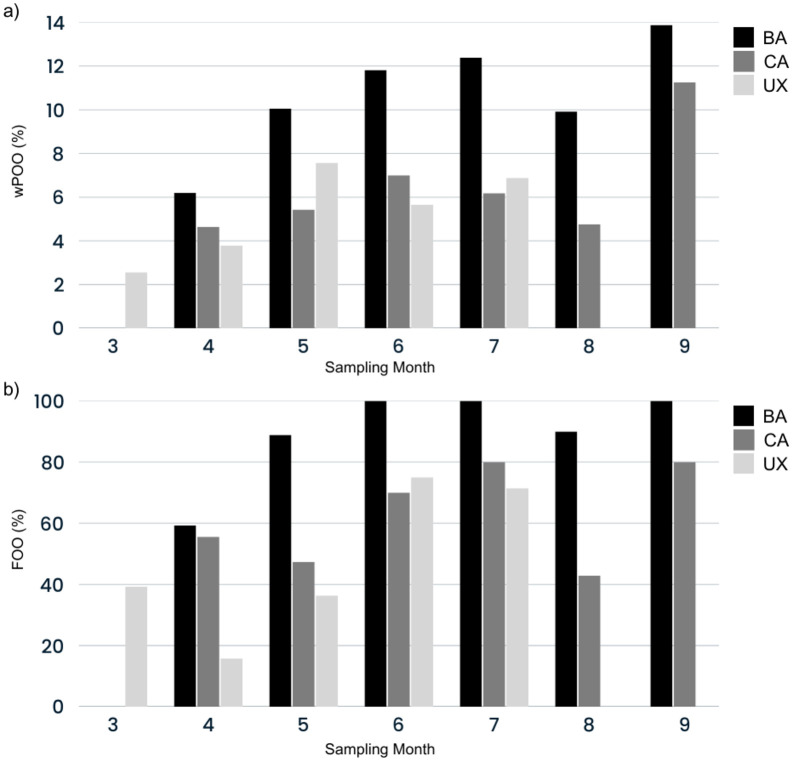
Monthly pest consumption by *Rhinolophus ferrumequinum* colonies in (a) wPOO (weighted percentage of occurrence) and (b) FOO (frequency of occurrence). Note that month 3 has data only for the UX colony, and months 8 and 9 have data only for BA and CA, respectively. BA: Balmaseda; CA: Caseda; UX: Uxue.

We found pests in 147 out of 237 samples. 84.34% were positive samples for pests in BA, 59.26% in CA and 39.73% in UX. In total, 50 identifications were pest species or genera (Supporting Information 1). We found 39 pest species in BA, 29 in CA and 22 in UX. Pest species were consumed throughout the entire season in every colony. The peak of pest consumption in BA coincided with the general pattern, while the second highest in CA occurred in June, followed by July. Finally, May showed the highest pest consumption in UX, followed by July and June (Fig 5).

We found 20 pest species in the core diets (Table 4). BA was the colony where more pest species were consistently consumed. It had 17 species in its core diet, followed by CA with 6 and UX with just 2 pest species. Most species were related to horticultural crops, fruit and nut trees, and some were among the 10 most consumed prey in some months. In CA, even if the main consumed prey species were linked to forestry (*Malacosoma neustria, Cydia fagiglandana, Thaumetopoea pityocampa*), others were associated with orchards and horticultural species (*Opogona sacchari* and *Agrotis segetum*).

**Table 4:** Pest species identified on the core diets (FOO > 5%) of *R. ferrumequinum*, their host species and their abundance in diet (wPOO). Values for individual colonies (BA: Balmaseda; CA: Caseda; UX: Uxue) reflect the proportion within each colony’s samples, whereas the total (TOT) represents the proportion across all samples combined, which may result in lower overall percentages.

Species	Pest of	TOT	BA	CA	UX
***Agrotis bigramma***	*Vitis vinifera, Zea mays, Medicago sativa, Spinacia oleracea, Lactuca sativa, Beta vulgaris, Cichorium sp., Borago officinalis, Brassica oleracea, Cynara cardunculus, Solanaceae*	0.09	0,25	0.00	0.00
***Agrotis exclamationis***	*Vitis vinifera, Zea mays, Medicago sativa, Spinacia oleracea, Lactuca sativa, Beta vulgaris, Cichorium sp., Borago officinalis, Brassica oleracea, Cynara cardunculus, Solanaceae*	0.14	0.34	0.05	0.00
***Agrotis ipsilon***	*Vitis vinifera, Zea mays, Medicago sativa, Spinacia oleracea, Lactuca sativa, Beta vulgaris, Cichorium sp., Borago officinalis, Brassica oleracea, Cynara cardunculus, Solanaceae*	0.19	0.32	0.05	0.19
***Agrotis puta***	*Vitis vinifera, Zea mays, Medicago sativa, Spinacia oleracea, Lactuca sativa, Beta vulgaris, Cichorium sp., Borago officinalis, Brassica oleracea, Cynara cardunculus, Solanaceae*	0.22	0.19	0.22	0.26
***Agrotis segetum***	*Vitis vinifera, Zea mays, Medicago sativa, Spinacia oleracea, Lactuca sativa, Beta vulgaris, Cichorium sp., Borago officinalis, Brassica oleracea, Cynara cardunculus, Solanaceae, Gossypium sp.*	0.38	0.68	0.40	0.00
***Aleimma loeflingiana***	*Quercus sp.*	0.38	0.07	0.31	0.81
***Archips xylosteana***	*Corylus avellana, Quercus sp.*	0.25	0.09	0.39	0.29
***Autographa gamma***	*Vitis vinifera, Medicago sativa, Spinacia oleracea, Lactuca sativa, Beta vulgaris, Cichorium sp., Borago officinalis, Brassica oleracea, alcachofa, cardo*	0.27	0.63	0.00	0.15
***Curculio elephas***	*Castanea sativa, Quercus sp.*	0.24	0.46	0.24	0.00
***Curculio glandium***	*Quercus sp., Corylus avellana*	0.24	0.49	0.05	0.15
***Cydia sp.***	Fruit trees*, Castanea sativa, Juglans regia, Quercus sp.*	0.20	0.45	0.11	0.00
***Cydia fagiglandana***	*Castanea sativa, Quercus sp.*	0.42	0.55	0.54	0.15
***Malacosoma neustria***	*Quercus sp.*	0.53	0.55	0.98	0.00
***Mythimna unipuncta***	*Oryza sativa, Zea mays*	0.12	0.35	0.00	0.00
***Noctua pronuba***	*Vitis vinifera*	0.10	0.27	0.00	0.00
***Opogona sacchari***	*Ficus sp.; Solanum tuberosum; Zea mays…*	0.85	1.95	0.47	0.00
***Pandemis heparana***	Pome fruit trees	0.08	0.19	0.05	0.00
***Peridroma saucia***	*Medicago sativa*	0.15	0.34	0.09	0.00
***Thaumetopoea pityocampa***	Conifers	0.44	0.63	0.44	0.22
***Tuta absoluta***	*Solanaceae*	0.23	0.05	0.20	0.47

Certain species have shown notable consumption over time, underscoring their importance. For instance, *Opogona sacchari* in BA was the most consumed species, with a total wPOO of 1.95% and a FOO of 53%. Its monthly consumption exceeds 2.5% from May to July (Fig 4).

**Fig 6:**
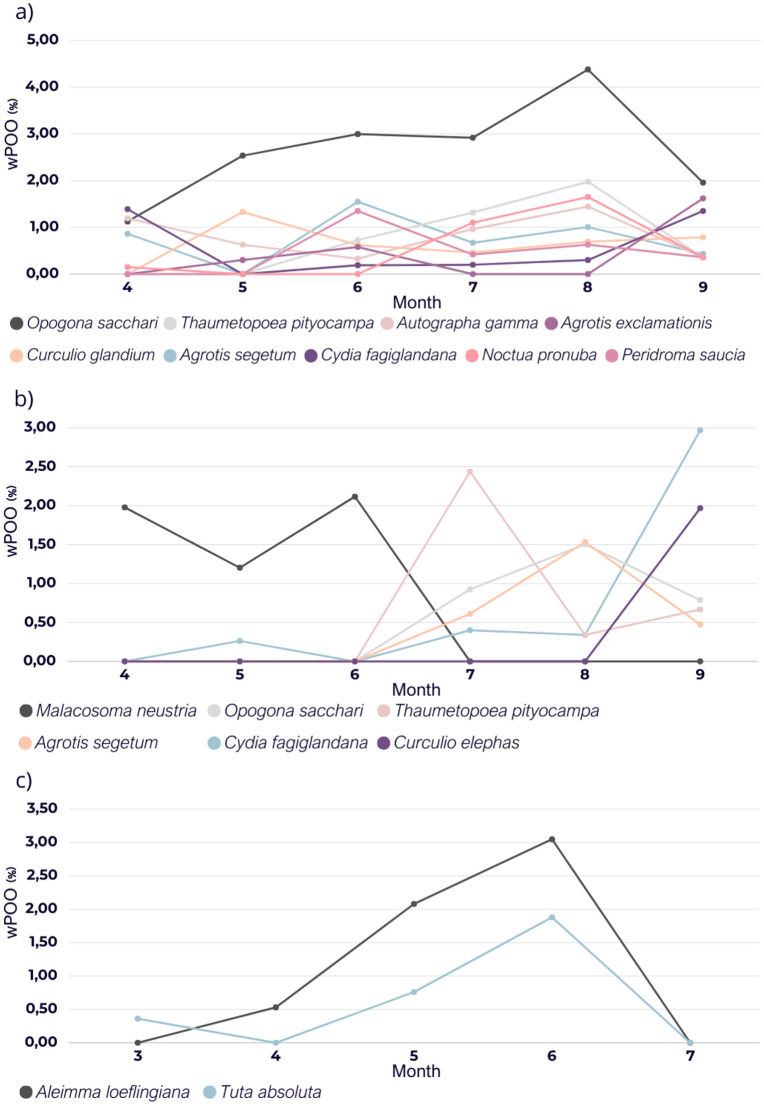
Consumption of pest species during the active season by *Rhinolophus ferrumequinum.* Species have been selected from the five most consumed species per week and colony. Consumption has been calculated as wPOO. **(a)** BA: Balmaseda colony, **(b)** CA: Caseda and **(c)** UX: Uxue.

Following the typical emergence pattern of insects, but especially that of pest species, the consumption of these prey also peaks during their periods of highest abundance (Fig 6; Gil et al. [49]). Some of the most consumed pests affect certain crops of commercial value, such as vineyards, diverse vegetables, and nut or fruit trees. It is the case of *Tuta absoluta* in UX, affecting different Solanaceae species; *Agrotis segetum* and other *Agrotis* species affecting vineyards, different orchard species or corn in CA and BA; or *Thaumetopoea pityocampa,* which affects conifers in CA, especially in BA, where conifer plantations are of great economic importance.

### Pest consumption estimates

Despite *R. ferrumequinum* exhibiting, on average, less frequent pest consumption (in terms of wPOO) than *R. hipposideros,* its larger size results in significantly higher overall consumption (mass of pests). The estimated consumption for *R. ferrumequinum* bat ranges from 1.21 tons (when c = 0.3) to 2.83 tons (when c = 0.7) of pests over five months, in contrast to the 0.38 to 0.88 tons consumed by *R. hipposideros*(values calculated for a 17,625 km² study area).

The highest pest consumption value for *R. ferrumequinum* was assessed in BA, with consumption ranging from 33.98 to 79.28 kg. This was followed by CA, which ranged from 10.37 to 24.21 kg, and by UX, which ranged from 8.39 to 19.58 kg. On the other hand, although individual pest consumption was higher in *R. hipposideros* (in terms of wPOO), their lower nightly intake resulted in much lower overall colony pest consumption. The colony with the highest pest consumption was ZA, with a range of 5.22 to 12.18 kg, followed by MA, with 4.61 to 10.76 kg, and then the smallest colony, CA, with a range of 0.52 to 1.21 kg. In general, pest consumption tended to be higher in areas with higher levels of urbanisation (Table 1; [47,48].

Based on the foraging range estimates of *R ferrumequinum* (approximately 5 km radius from the roost: [57–61]) and *R. hipposideros* (600 m radius in average: [62]), at the CA site, the foraging ranges of the two colonies are likely to overlap, potentially enhancing local pest suppression through combined predation.

## Discussion

Our results confirm that these horseshoe bat species consume a wide range of agroforestry pest insects [4,50]. Some of these pests are associated with economically important crops such as vineyards, orchards, and diverse vegetables. However, we emphasize that the total number of pest species consumed is less significant than the subset of taxa more consistently detected across samples. While many prey species appear only sporadically or as ‘accidental’ detections, others—though representing a small absolute percentage of the total diet—exhibit higher relative frequencies and constitute what is described as the species’ “core diet” [50]. In the context of the high taxonomic diversity captured by DNA metabarcoding, a weighted Percentage of Occurrence (wPOO) of 5% reflects a stable dietary component compared to the vast majority of prey taxa that appear at trace levels (wPOO < 1%). Consequently, these more frequent detections identify the pests that are most systematically integrated into the foraging ecology of these bat species.

The insect taxa comprising the core diet of bats are relevant because they often undergo significant outbreaks, allowing bats to benefit from their population peaks and increases in density [16]. Insectivorous bats show notable adaptability, allowing them to exploit localized insect hotspots and episodic outbreaks [11,12,14,15,63,64]. This dietary adaptability enables wide-dietary-spectrum bats to contribute to the suppression of various pest taxa, providing a notable ecosystem service.

The enormous seasonal variability in pest consumption that we have found, which likely responds to pest outbreaks, is a significant finding. This pattern has already been described in other studies and aligns with the typical seasonal insect consumption pattern of bats [3,14,15]. Additionally, the variability in pest consumption may be influenced by surrounding habitats, which likely shape local arthropod diversity. Furthermore, the discovery of species linked to rice crops in CA suggests that bats’ impact can extend beyond their foraging areas [17]—up to 4.2 km from the roost [62]— thanks to the river’s connectivity, as rice croplands are located kilometres downstream from the maternity roost (maps available at [47]).

Pest consumption in the CA and UX regions was remarkably lower than in the northern colonies. This difference can be attributed to the diverse natural habitats nearby, with a high arthropod diversity. Bats in these areas have access to large rivers with well-preserved riparian forests near their colonies, providing them with a substantial supply of riparian and generalist prey [47,48]. Furthermore, the predominant agricultural model in the region is intensive, in which farmers presumably manage the major pest species affecting these crops, with specific or generalist pesticides. Pesticide use could also explain the lower levels of pest consumption in these areas.

The estimates we have made regarding insect consumption by bats are not exact. On the one hand, the total amount of insects consumed by bats each night can vary significantly, ranging from 30% to 100% of their body weight (e.g., [51,52,55]). Additionally, consumption rates can differ between species and fluctuate with the seasons, as our results have demonstrated. Moreover, there are no precise population estimates for these bat species. Despite these limitations, our estimates provide a valid range that helps us quantitatively assess these bats’ impact on agriculture and highlights the importance of frequent and abundant bat species [28].

A study by Aizpurua and Alberdi [65] examined the impact of European bats on pest arthropods. They found that *R. ferrumequinum* and *R. euryale* are among the largest consumers of pests per individual bat. While *R. ferrumequinum* has a broader diet, which results in a lower pest incidence, its larger body size enables it to consume a significant quantity of pests, making its overall pest consumption comparable to that of more specialised species. Similarly, our results show that although the average weight proportion of pest species (wPOO) was lower in *R. ferrumequinum* compared to *R. hipposideros*, the total weight of pests consumed was greater in *R. ferrumequinum*. However, *R. hipposideros* locally exerted intense predatory pressure on pest species such as *Opogona sacchari* during its peak occurrence. Overall, these findings, together with those of Aizpurua and Alberdi [65], indicate that horseshoe bats play a particularly important role in local pest suppression within the study area and are likely important contributors to pest control within the broader European bat community.

While the pest consumption of individual bat species is interesting, the interactions among different bat species within the same environment are even more intriguing. Research indicates that various bat species belonging to different guilds —and therefore with varying adaptations of foraging/preferences— feed on similar agricultural pest species, including *Agrotis sp., Autographa gamma, Mythimna sp., Noctua sp., Pandemis heparana, Peridroma saucia, Thaumetopoea pityocampa,* among others (e.g., [3,4,14,66], present study). This shared predation implies that when agricultural pests —or other insect outbreaks— are present, bats in the area tend to feed on them consistently. Consequently, bat communities can play a vital role in controlling pest outbreaks.

We would also like to emphasise the potential of bats to detect outbreaks of pest species, including those that are unknown or those for which no control measures are in place. A notable example in this study is *Opogona sacchari,* a moth species that affects more than a hundred crop types (EPPO Global Database). Although this species is listed as an agricultural pest in Portugal and France, it was previously unknown in continental Spain. Its significant presence in the diets of both *R. ferrumequinum* and *R. hipposideros,* in BA and ZA, respectively, indicates that it is already widespread and may impact various agroforestry crops in the study area. Therefore, we want to highlight the value of bats as trackers of present and future entomofauna and pest controllers, as they can detect and consume species that may go unnoticed or unmonitored by conventional human surveys. [67]. Hence, common bat species can help control and mitigate ecosystem imbalances caused by factors such as changes in farming practices, landscape disturbances, and climate change, offering a hopeful perspective on their ecological impact.

Finally, we would like to emphasise the importance of protecting bats’ natural roosts, installing artificial roosts, and maintaining landscape heterogeneity, as these are crucial for preserving their ecosystem services [8]. Roost conservation is especially vital for cave-dwelling species, as underground habitats are vulnerable to disturbances and require regulation and protection [68,69]. Artificial roosts can support bat populations in areas with limited natural shelters, such as agroecosystems, enhancing their ecosystem contributions [7,70–72]. Additionally, promoting habitat connectivity benefits arthropod populations and improves bat foraging opportunities [73]. These results also underscore the need for enhanced public education on the ecological importance of bats and their ecosystem services, as increased awareness and positive perceptions can support conservation actions and long-term population stability (e.g., [74,75]).

## Supporting information

Supporting information 1 Pest identifications in *R. hipposideros* and *R. ferrumequinum* diets and their per-sample wPOO values. (XLSX)
