# Supplementary material for: Pest consumption by common bats in diverse landscapes
Source: PLoS One. 2026 Jun 17;21(6):e0347853. doi: 10.1371/journal.pone.0347853 (PMC13274907; doi:10.1371/journal.pone.0347853)
Supplement: Attachment — Submitted filename: SecondReview_PONE-D-25-25129.pdf [file pone.0347853.s003.pdf]

Journal: PLOS ONE
Manuscript ID: PONE-D-25-25129
Second Review

Pest consumption by common bats in diverse landscapes

This manuscript explores how bats provide essential ecosystem services in the form of insect pest consumption. Using *Rhinolophus* bats as focal species, the authors used insect DNA found in fecal samples to estimate the impact bats were having on insect pest species in the region.

Main Comments:

- The authors did a diligent job on addressing the comments and critiques from the first review. The manuscript has greatly improved, and I only have a few small recommendations. The manuscript is greatly improved and provides much better context for the work and findings.

Specific suggestions and remarks

Introduction

75-76: Consider separating into two paragraphs with the species info in the next

Methods

88-89: Be more specific as to where this study is occurring; state the country.

91: Expand the figure description. What is the inset map? What is it focusing in on?

122-128: Move the ethics statement up further
