# Supplementary material for: Pest consumption by common bats in diverse landscapes
Source: PLoS One. 2026 Jun 17;21(6):e0347853. doi: 10.1371/journal.pone.0347853 (PMC13274907; doi:10.1371/journal.pone.0347853)
Supplement: Attachment — Submitted filename: Response to reviewers.docx [file pone.0347853.s002.docx]

RESPONSE TO REVIEWERS

Dear Prof. Mark A. Davis,

Please convey our sincere appreciation to the Reviewers for their thorough and constructive evaluations. We have carefully revised the manuscript in line with their recommendations, as detailed below and in the marked version of the manuscript. In a few instances, however, we respectfully disagree with the suggested revisions, and we provide a clear justification for our position in each case.

**Reviewer #1: Journal: PLOS ONE**

This manuscript explores how bats provide essential ecosystem services in the form of insect pest consumption. Using *Rhinolophus* bats as focal species, the authors used insect DNA found in fecal samples to estimate the impact bats were having on insect pest species in the region.

Main Comments:

• The introduction introduces the material well, but there are parts where the language is vague and there are not good connections between paragraphs. I recommend spending more time linking the concepts to why the focal species is a good model a bit more. Additionally, spend more time explaining on how insects are considered pest species in the region and connecting it to your focal species.
• Overall there is excellent information herein, but there is a lot of vague language used and there needs to be more time spent connecting concepts together.

• There are some changes needed for the tables and figures to make them cleaner and easier to understand. Always remember that the reader should be able to just read the captions and know what the figure is about without ever seeing it.

• Because the reader may be unfamiliar with the region, I recommend including a map with where the site are located/where samples were collected.

• The abstract states that the study is during the breeding season, but does not explain well more of the life history of the focal species. The study covers 7 months, which may encompass the breeding season and then some. More care should be given introducing the focal species and what they are a good model for this study (which I do think they are).

Dear Reviewer,

We sincerely thank you for your thorough and constructive evaluation of our manuscript. Your comments have greatly contributed to improving the clarity, structure, and overall quality of our work. We have carefully addressed each of your points and revised the manuscript accordingly. Below, we provide detailed responses to all suggestions, indicating where modifications were made in the revised version.

We substantially revised the Introduction to improve conceptual flow, clarify the framing, and reinforce the relevance of the focal species. We expanded the explanation of why horseshoe bats represent an appropriate model for assessing bat-mediated pest suppression.

We revised the text extensively to eliminate vague wording and strengthen conceptual connections across sections. Many sentences and paragraphs were rephrased for precision and clarity, following your suggestions.

We revised all figure and table captions to be more detailed and self-explanatory and applied formatting adjustments to the tables and axis labels as recommended. Explanations of units and terminology were added where needed.

In addition, we have added a new map illustrating the locations of all sampled colonies (new Figure 1).

**Specific suggestions and remarks**

**Introduction**
37-38: Reduce sentence length “…feed on a wide variety of arthropods, including many pest species such as …”

**Answer**: OK, we rephrased the sentence. (lines 36-37 of the revised manuscript)

40: Vague language; “This makes bats vital…”

**Answer**: OK, we rephrased the sentence. (lines 38-39)

42: Bats can offer; leave the potential open ended so that it gives the reader the sense that your work is adding to the collection of work

**Answer**: OK, we included “can” in the sentence (line 40)

46: Plasticity of what? Be more specific and then give examples of those.

**Answer**: OK, we rephrased the sentence. (lines 43-45)

51-52: Avoid vague words such as “this”. Consider rephrasing “Because of bats ability to adjust their diets and foraging habitat, they are highly effective pest suppressors…”

**Answer**: OK, we rephrased the sentence. (lines 49-51)

60-61: Inappropriate to call other studies “run of the mill”. It gives the wrong impression about their value

**Answer**: We rephrased the paragraph and removed this phrase (next point). (lines 52-65)

60-67: I would consider reframing this paragraph to focus on how fecal DNA is a great tool to understand bat diets, and thus the pests they consume.

**Answer**: OK, we changed the paragraph to emphasise the role of metabarcoding. (lines 52-65)

68-79: These two paragraphs do not connect to one another well. Maybe spend time on why it is important to use abundant and widely distributed species can help **Answer** the question, and then go into your focal species as a model.

**Answer**: OK, following this suggestion we changed the previous paragraph about the importance of abundant and common species to link both paragraphs. (lines 66-83)

**Methods**

82: Be more specific as to where this study is occurring. It may be worth including a map of the region, and where samples were collected.

**Answer**: We included a figure with the location of the sampled colonies. (new Figure 1)

90-99: Inconsistent with time periods. Some state the week collection occurred, others just the month/day. Be consistent.

**Answer**: OK, we changed the time periods to always include the Julian week and the month (lines 97-103)

100-105: You introduce “OUT”s here, but do not explain what it is. It is explained later on, but currently the reader does not know what OTUs are. I suggest either expanding this section, or moving it further down to be aligned with the description of OTU.

**Answer**: OK, we added the explanation of “OTU” in the current sentence. (lines 117-118)

145: If it is unpublished, how are the data accessible?

**Answer**: At the current moment, it has already been published, so the reference “Aldasoro et al., 2025b” has been added. (line 159)

151: If focusing on these uncertainties, spend more time in the intro about the specifics.

**Answer**: Yes, the new version of the Introduction addresses this issue (lines 52-65).

156-170: It says there is a formula, but I do not see one written out.

**Answer**: Sorry about that; it looks like the format change caused it to disappear. We added the formula (line 170)

**Results**
174: Were the samples pooled? Or were they tested individually? Is this proportion just the samples that tested for any pest species? What is the proportion of pest species within the positive samples?

**Answer**: We included an explanation of sample collection in the ‘Sampling Bat Droppings’ section (lines 106-108). All the samples included in the study were positive for arhropod prey. The reported proportion refers to the fraction of faecal (community) samples that tested positive for any pest species.

192-193: Linked how? Be more specific.

**Answer**: OK, we rephrased the sentence. (lines 209-212)

193-195: Species of bat or of pest insect? Be more specific and how they are linked to the plant species.

**Answer**: OK, we rephrased the sentence (lines 210-211)

Since these are pest species of agricultural crops, their link / association indicates that they also pose a threat to these specific cultivars.

230-232: How do we, the reader, know that is the usual time pest species emerge? What does “typical emergence pattern” mean? Is it species specific?

**Answer**: While the Integrated Pest Management Guides do not provide detailed temporal abundance data, they do indicate the peak seasons for each pest species, which aligns with our observations of peak bat consumption. We have added the citation to the text: (Gil et al. 2014). (lines 47-48). We have also revised the corresponding statements in the manuscript to ensure they accurately reflect the resolution of our data while maintaining alignment with the information available in the literature.

243-245: State earlier species general differences, such as their sizes.

**Answer**: OK, we included a short sentences stating these differences in the introduction. (lines 74-79)

251-252: Why do they have lower energy costs? If this is an assumption because of size, I suggest removal of the sentence. If it is supported in the literature, then cite the sources.

**Answer**: No, you are right. To our knowledge there is not any information about that. We rewrote the sentence to make it clearer. (lines 269-271)

**Discussion**
257: That some species of horseshoe bats.

**Answer**: OK, we changed the sentence. (line 281)

257-258: Get rid of “because of their remarkable adaptability”. Suggest it later in the discussion if appropriate.

**Answer**: OK, removed.

260: Remove “in this study”

**Answer**: OK, removed.

261-263: Combine sentences; “…resulting in few total occurrences, while other insect species are heavily consumed and form what is known as “core diet”.”

**Answer**: OK, we combined the sentences as suggested (lines 285-286)

263: “These” is vague; “The insects comprising the core species…”

**Answer**: OK, we changed the sentence as suggested (lines 287)

265-266: Explain more the adaptability you document ahead of the ending sentence such as “Because we documented bats doing x,y,z, we argue that they have an adaptable diet and thus…”

**Answer**: OK, we included a previous sentence pointing out this adaptability. (lines 289-291)

270-271: This is not really what your study looked at, but your study does suggest it. Propose the ideas as potential hypotheses, not as fact.

**Answer**: OK, we agree and changed the sentence accordingly. (lines 296-297)

278-281: This sentence seems out of place compared to the topic sentence.

**Answer**: OK, we agree and changed that sentence to the end of the previous paragraph (lines 297-300)

299-305: I think this paragraph, while interesting, is not necessary and could be removed or incorporated elsewhere.

**Answer**: Here we disagree. In fact, in our opinion, one of the most interesting findings on the trophic ecology of insectivorous bats during the last years is that bat species belonging to different guilds —and therefore with different foraging adaptations/preferences— tend to converge, opportunistically exploiting the same insect outbreaks (e..g. Garin et al. 2019; Aihartza et al. 2023; Aldasoro et al. 2025a and 2025b...). So, we would keep this paragraph if the editor agrees. (lines 326-334)

**Tables and Figure Captions**

Table 2: Add more to the table caption including writing out the full site names and species names.

**Answer**: OK, we added the required information to the table caption. (lines 186-189)

Table 3: Use decimal points rather than commas.

**Answer**: OK, changed to decimal points. (lines 223-227)

Table 4: A bit confusing; the total diet is less than individual sites. Explain more in the caption so that the reported values make more sense.

**Answer**: We agree that this can appear confusing. The weighted percentage of occurrence reflects the proportion of occurrences within each site. For individual colonies, a given pest group can represent a relatively high percentage because it is calculated only from that colony’s samples. When all colonies are combined, the total number of samples increases substantially, so the same pest group represents a smaller proportion in the overall dataset. We clarified this in the table caption to make the values easier to interpret (and also in Table 3). (lines 257-260 and 223-227)

Figure 1: Explain how sampling week is in relation to the week of the year somewhere.

**Answer**: OK, we added month names under the Julian weeks (Now Figure 2)

Figure 2: Spell out full bat species name. The metric is needed (%?) for the wPOO. What are the units?

**Answer**: OK, we added the full species name to the figure caption and added (%) to the y axis. (Now Figure 3) (lines 216-218)

Figure 3: Units needed on (a); Kg of what? Put more on axis label

**Answer**: OK, we added (%) to the y axis in (a) and “Pest Kg” to (b). (Now Figure 4)

Figure 4: Is this for both species or just one? State it in the figure caption.

**Answer**: OK, we added “*Rhinolophus ferrumequinum*” to the caption. (Now Figure 5) (lines 244-246)

Figure 5: Spell out full bat species name. Add units on axis.

**Answer**: OK, added the full species name to the figure caption and added (%) to the y-axis. (Now Figure 6) (lines 254-256)

**Reviewer #2:**

The manuscript, “Pest consumption by common bats in diverse landscapes”, details an important narrative of the ecosystem service that bats provide as pest consumers. Quantifying the abundance and diversity of pests that bats consume in varying agricultural settings throughout a season plays an important role in promoting bat conservation and overall ecological health. This manuscript supports this foundational knowledge by studying two common & abundant species in Spain and describing their pest consumption throughout the season. While the manuscript makes several claims without citations or data to support them, with further citations and specifications, this manuscript will benefit the body of literature well.

I think my biggest issue with this manuscript is that it makes the claim that the seasonality in consumption of different pests follows the pulses in pest abundance associated with hatches. However, there are no citations or data that support the seasonality of pulses in pest abundance. Without supporting data to show that the peak in species-specific pest abundance mirrors the peak in species-specific consumption, I don’t think you can make this claim. I believe that this is a highly-probable theory, but the diet data that you are analyzing doesn’t support this claim until you add additional data about seasonality of the pest abundance.

Additionally, you also make the claim that lower pest abundance comes from sites that have more diverse and natural surroundings, however you don’t provide any data to back this claim up. This seems like an easily quantifiable fix with land use imagery to quantity the amount of natural area to anthropisation around each site, but without the data to support this- its an unsubstantiated claim in the paper. Detailed in the comments below are more instances where more specifics are needed or claims are quite broad.

Dear Reviewer,

We sincerely thank you for your thorough and constructive evaluation of our manuscript. Your comments have greatly contributed to improving the clarity, rigor, and overall quality of our work. We have carefully addressed each of your points and revised the manuscript accordingly. Below, we provide detailed responses to your specific comments, indicating where modifications were made in the revised version.

Regarding your primary concern about the seasonality of pest consumption, although fine-scale temporal abundance data for pest species are not available, the Integrated Pest Management Guides provide information on the general peak seasons for each pest species. These documented seasonal peaks align with the periods in which we observed elevated bat consumption (Gil et al., 2014). The manuscript has been revised to clarify that our findings are consistent with these documented seasonal trends while accurately reflecting the resolution of our data.

Concerning landscape effects on pest abundance, this study builds on prior work (Aldasoro et al., 2025a, 2025b), which quantified land use around each colony. Relevant information has now been added to Table 1, detailing the proportion of natural versus anthropogenic habitats, thereby providing context for observed differences in pest abundance across sites.

**Specific Edits:**

Line 27: “they can go one step beyond human capabilities” – Please be specific about what you mean here.

**Answer**: OK, we rewrote the sentence to be clearer. (lines 24-26 of the revised manuscript)

Line 29: “several tons of insects” – Give a range of values that you have found here.

**Answer**: OK, we changed the sentence (line 28)

Line 50: “frequently consume insects from outside their hunting grounds” – I would say that many bats do not have specific hunting grounds, but forage opportunistically as you mention. I think just rewording this phrase would be best.

**Answer**: OK, we rephrased the sentence to include the opportunistic option. Nevertheless, Arrizabalaga et al. (2015) reported that the source-habitat of many of the main prey consumed by Mediterranean horseshoes didn’t fit their preferred foraging habitats, thoroughly studied by radio-tracking in the very same study area. (lines 46-49)

Line 56: “The larger the population, the greater the collective consumption of pests” – This seems like an assumption to me and I could think of many ecological scenarios where abundance of bats does not correlate with pests consumed. I would rephrase this to accommodate some of this uncertainty.

**Answer**: OK, we rewrote the sentence. (lines 68-70)

Line 60: “Run-of-the-mill” – This seems a little derogative to the studies mentioned- I would choose a different descriptor.

**Answer**: OK, we rephrased the paragraph and removed this phrase. (lines 52-65)

Line 65: What method have these studies used to estimate pest consumption? You refer back to this later, so I think it would be helpful to be specific here.

**Answer**: OK, we have moved this paragraph, but have included the estimation methods. (lines 57-59)

Line 77-79: I think a clearer description of research question, study objective, and study importance would be beneficial here. The last sentence just feels tacked on without really incorporating it into the study importance. Just rephrasing this would create a much stronger introduction

**Answer**: OK, we rephrased it. (lines 79-83)

Line 83: Double check your species names here- I think you can start using abbreviations for both species. This happens several times throughout the paper including with the pest species, but I am not going to list them all.

**Answer** We agree that abbreviations can be used after the first mention of each species. In several sections, we spelt out the full species names because journal conventions often require doing so at the beginning of a new section. We are fully open to adjusting this formatting and will follow the editor’s final guidance on preferred usage.

Table 1: Population sizes in colonies vary quite a bit over the course of the year as juveniles are born. Are these populations sizes pre- or post- birth pulse in abundance?

**Answer**: These population sizes represent an approximation of the pre-birth colony size. We have clarified this in the table caption to avoid any misunderstanding. (lines 91-95)

Line 97: “Up to 24 samples of 4-6 pellets” - How did you decide on how many samples per session? How did you did you decide on which pellets to pick up? Randomly? Off the top? Largest? Most whole pellets?

**Answer**: We aimed to collect the freshest and most intact pellets to ensure DNA quality. In mixed colonies of *R. ferrumequinum* and *M. emarginatus*, we selected the larger pellets because they most likely belonged to *R. ferrumequinum*. The number of samples per session followed sampling efforts used in previous studies, which guided our choice of collecting up to 24 samples of 4–6 pellets each. We rephrased that paragraph. (lines 106-111)

Line 101: How many samples were considered contaminated from the 413 and 377 samples?

**Answer**: The samples belonging to *R. hipposideros* were not contaminated, but around a third of the *R. ferrumequinum* samples were contaminated. We included that information. (lines 113-116)

Line 102: Here you say you are analyzing them based on sampling month, but some of your figures are divided up by sampling week? Is that just for R. ferrumquinum? I’m a little confused about this part.

**Answer**: Yes, the shift to monthly analyses applies only to *R. ferrumequinum*, as the removal of contaminated samples substantially reduced the weekly sample size. For *R. hipposideros*, the sample size remained sufficient, so the weekly resolution was retained for all analyses. We added a clarifying sentence at the end of the paragraph to make this distinction more explicit. (lines 119-120)

Line 142: How did you decide what was potential prey and what wasn’t?

**Answer**: As described in the previous section, OTUs were classified as potential prey when they corresponded to arthropod taxa occurring within our study area. For OTUs with multiple possible assignments, we manually reviewed and refined the taxonomy by considering pairwise identity and known species distributions to determine the most accurate classification.

Line 154: I would love to see a map of site locations and this general region in Spain- I think this would help delinate some of the later population size estimates.

**Answer**: We included a figure with the location of the sampled colonies. (new Figure 1)

Line 156: You outline your variables here, but I never see an actual formula about how you calculate these together?

**Answer**: Sorry about that; it looks like the format change caused it to disappear. We added the formula (line 170)

Line 160: It would be good to know the where the 30% comes from in these studies as compared to the 70% in c1. Why such a big difference between the two? Are the studies for c2 also during pregnancy and lactation? Or males? If you just chose the min and max values from the literature, I would just state it as such.

**Answer**: We selected these values to provide a range encompassing the variability reported in the literature. The proportion of pest consumption can differ widely depending on species, season, or reproductive status, so the 30% and 70% values were chosen as approximate lower and upper bounds, respectively. We stated that in lines 175-176)

Line 163: “Basque Country of Aihzartza (2001)” – Reword: it sounds like there is a country in Basque named Aihzarta?

**Answer**: OK, we rephrased it to make it clearer. (lines 177-178)

Table 3: I understand that you are using wPOO here, but it feels a little nebulous to me. If the max that a wPOO can be is 100% (where they only eat one thing), then 8.5% of insects that R. hipposideros eats is a pest seems rather low?

**Answer**: Although 8.5% may seem low, this value represents the mean consumption across the entire community and over five months. Considering this, a 10% contribution of pest species to the total diet is not negligible.

Figure 2a: I would reduce the y-axis range down to 1500 wPOO. Since there is only one main high point in that range, it would be more easier to read the smaller values.

**Answer**: OK, changed the y-axis to a maximum of 15 (now Figure 3a)

Line 232: I think this is a key point in your paper, but it would be a lot stronger to have data to back this up. Does the Integrated Pest Management Guides of the Spanish Government have temporal data to cite for an abundance comparison with the peak consumption? Otherwise, you are stating this with no citation or evidence to back the claim up.

**Answer**: While the Integrated Pest Management Guides do not provide detailed temporal abundance data, they do indicate the peak seasons for each pest species, which aligns with our observations of peak bat consumption (cited accordingly as Gil et al. 2014). (lines 247-248)

Line 245 & 247: You now use the common name here- I would stick to either Latin name or common name.

**Answer**: OK, we changed to the Latin names. (lines 262-266)

Line 248: I think the map part may help a bit here, but how far of a spatial scale would the 79.28kg eaten be covering? An estimate of the home range of the colonies would be beneficial here. Would you expect this estimate to vary between colonies? Are the ranges in colonies overlapping?

**Answer**: The foraging ranges of the species are approximately 5 km radius for *R. ferrumequinum* and up to 600 m radius for *R. hipposideros*. In the CA site, the foraging ranges of the two species colonies would overlap, which may influence local pest suppression.

We included a short paragraph highlighting this information. (lines 275-279)

Line 254: This is the first time that you mention anthropisation in the paper. How did you calculate this? Which sites are higher or lower in terms of anthropisation?

**Answer**: You are right that this is the first mention of anthropisation in the manuscript. This study builds on two previous papers (Aldasoro et al. 2025a, 2025b) that provided detailed land-use maps, showing the degree of urbanisation and cropland surrounding each colony. To ensure the present manuscript is self-contained, we have now added the percentages of cropland/orchard and urbanised area within a 3-km buffer around each site to Table 1.

We also revised the sentence to reduce ambiguity. (lines 273-274)

Line 257: Based on what you just presented, it seems like they eat more than “several” agroforestry pests?

**Answer**: OK, we have replaced the word “several” with “a wide range of” to more accurately reflect the diversity of agroforestry pest species detected in the bats’ diet. (line 281)

Line 264 &267-268: Again, I think your work would be a lot stronger if you had data to compare the peak outbreaks of pests with the peaks in consumption and showed that bats are moving from one pest to the next and are truly following the varying peaks in abundance. Right now, you just show us that consumptions changes throughout the season without proving to us that it follows peak abundance. I get that you may not have data to prove localized abundance, but even literature supporting the timing of these peaks would be strengthen your work.

**Answer**: As noted in our response to an earlier comment, although the Integrated Pest Management Guides do not provide fine-scale temporal abundance data, they do identify the general peak seasons for each pest species. These documented seasonal peaks are consistent with the periods in which we observed elevated bat consumption. We have revised the corresponding statements in the manuscript to ensure they accurately reflect the resolution of our data while maintaining alignment with the information available in the literature. (lines 293-294)

Line 269: “typical general” – I think you only need one of these words here.

**Answer**: OK, we corrected it. (line 296)

Line 274: Again, a map to see these rivers would be great. I also want to emphasize again that you haven’t provided us any context or data to differentiate between sites in the amount of “diverse natural habitat” nearby. Maybe in Table 1- you could put a percentage of natural habitat vs anthropogenic habitat? Its hard to know if you consider a pine plantation as natural or anthropogenic otherwise or what the difference is between Mediterranean bush vs shrubland?

**Answer**: As explained above, the landscape analyses were done in two previous papers (Aldasoro et al. 2025a, for *R. hipposideros*), where the maps are available, including the data sources used to build them (that information has been added at line 300). Besides, all pine plantations are marked as "forest" in the SIGPAC database used to build the maps, even though all of the pine plantations are anthropogenic in the area.

Line 279-281: “bats’ impact can exceed their foraging areas”- How do we know they aren’t foraging down there? Are the insects moving to the bats or vice versa? Or do the rice pests utilize other plants as well?

**Answer**: The closest rice plantations are approximately 15 km from the CA site, a distance far beyond the known foraging range of *R. hipposideros,* which —as far as we know— does not forage further than 3 km from the roost. Although we cannot entirely exclude the possibility that these pest species may also use other host plants in the surrounding landscape, the most plausible explanation—given the river connection and prevailing wind dynamics—is that the insects were transported toward the colony rather than the bats foraging near the rice fields. (lines 298-302)

Line 297: “horseshoe bats are among the most important bat species for pest suppression” – This seems like a bit of a stretch to me for the subject of this paper. How do we know that other species do not eat higher rates of pests? Are they the biggest bat in the region in which case, you could rephrase this comment? Maybe caveat it with “Given that horseshoe bats are one of the most common and can be one of larger-bodied bats in the region, …”

**Answer**: Thank you for the comment. The statement was based on the findings of Aizpuru and Alberdi (2020), who showed that horseshoe bats are among the largest consumers of pest species per individual across Europe. However, we agree that the original phrasing may appear too strong or generalised. We have therefore reworded the sentence to soften it, better reflect the evidence and to avoid overstatement. (lines 323-325)

Line 314: “one step beyond human capabilities” – Again, I feel like this phrasing is a little weird. I would reword since I can see what you are saying here.

**Answer**: OK, we rephrased the sentence to make it clearer. (lines 341-343)

Line 326: I also think a line in here about education on the importance of bats and their ecosystem services within the public would also be important for maintaining bat populations and positive bat sentiments.

**Answer**: Thank you for the suggestion. We agree that highlighting the importance of public education is essential for strengthening bat conservation and maintaining positive public attitudes. We have now incorporated a sentence addressing this point. Specifically, we mention that bat conservation plans benefit from improved communication strategies and increased public acceptance, which are crucial for long-term population stability. (lines 355-357)
