# Supplementary material for: Pest consumption by common bats in diverse landscapes
Source: PLoS One. 2026 Jun 17;21(6):e0347853. doi: 10.1371/journal.pone.0347853 (PMC13274907; doi:10.1371/journal.pone.0347853)
Supplement: Attachment — Submitted filename: RESPONSE_TO_REVIEWERS_auresp_2.docx [file pone.0347853.s004.docx]

**RESPONSE TO REVIEWERS**

Dear Dr Davis,

We thank you and the reviewers for the thoughtful evaluation of our revised manuscript. We are pleased to note the positive feedback regarding the improvements made to the paper. We have addressed the remaining points regarding geographic clarity, terminology, and ecological nuance.
Below, we provide a point-by-point response to the comments provided by the Editor and the Reviewers.

**Reviewer #1: Journal: PLOS ONE**

This manuscript explores how bats provide essential ecosystem services in the form of insect pest consumption. Using *Rhinolophus* bats as focal species, the authors used insect DNA found in fecal samples to estimate the impact bats were having on insect pest species in the region.

Main Comments:

• The authors did a diligent job on addressing the comments and critiques from the first review. The manuscript has greatly improved, and I only have a few small recommendations. The manuscript is greatly improved and provides much better context for the work and findings.

Dear reviewer,

We would like to sincerely thank the reviewer for their positive assessment and for the time spent evaluating our revisions. We are pleased that the added context and the clarifications regarding our methodology and findings have strengthened the manuscript. We have carefully addressed the remaining minor recommendations below to further refine the paper's clarity and nuance.

Specific suggestions and remarks

**Introduction**

**75-76:** Consider separating into two paragraphs with the species info in the next

**Answer:** OK, we agree and have divided the text into two paragraphs. (lines 86-87)

**Methods**

**88-89:** Be more specific as to where this study is occurring; state the country.

**Answer:** OK, added (line 106)

**91:** Expand the figure description. What is the inset map? What is it focusing in on?

**Answer:** OK, we have expanded the figure caption to provide better geographic context. We have clarified that the main map shows the specific study sites (colour-coded by species), while the inset map depicts the Iberian Peninsula. (lines 109-113)

**122-128:** Move the ethics statement up further

**Answer:** OK, we have relocated the Ethics Statement. It is now the first subsection within the "Materials and Methods" section (lines 98-104)

**Reviewer #2: Journal: PLOS ONE**

Thank you for your revision & thoughtful responses to reviewer comments. I believe that your manuscript is stronger because of these revisions. There are still some grammatical and spelling errors throughout the manuscript. Mainly this is a topic with a lot of nuances and I have listed out a couple spots below where I feel the nuance may still need to be addressed.

Dear reviewer,

We are very grateful for your positive feedback and for the recognition of the improvements made to the manuscript. We fully agree that the ecological implications of dietary studies require a high degree of nuance to avoid over-interpreting the data. Following your advice, we have carefully refined the language in the sections identified to better reflect these subtleties. Additionally, we have performed a comprehensive linguistic review to eliminate remaining grammatical and spelling errors.

**Line 171:** I would organise these descriptions based on the order they appear in your equation. Also your equation uses ‘pop’ and your description uses ‘pop size’ – these should be identical. I would also point out that you are using a population estimate from 2001 which is 25 years ago. Do you expect these population estimates to have changed at all? You also report that ‘other studies’ report similar population sizes between the two, but don’t cite these studies.

**Answer:** We have addressed each point as follows:

- We have reorganised the variable descriptions in the text to follow the exact order in which they appear in the equation. We have also unified the terminology, replacing "pop" with "pop size" in the equation to maintain consistency throughout the manuscript (lines 200-217).
- While the primary baseline for these species in the Basque Country was established by Aihartza (2001), we acknowledge the reviewer's concern regarding the age of this data. However, our research group has conducted continuous, long-term monitoring of the known colonies in the study area up to the present day. These ongoing observations suggest that while local fluctuations occur, the overall population scale has remained relatively stable over the last two decades. We have updated the text to clarify that the 2001 baseline was supplemented and validated by our recent field data (lines 211-212).
- We have added the missing citations (de Paz 2007; Migens 2007) to support the use of similar population estimates for both *R. ferrumequinum* and *R. hipposideros*. (line 215)

**Line 287:** I still feel a little conflicted about something that occurs in 5% of the total samples across all colonies being considered as “heavily consumed”. I understand that you have a citation to support this, but to me it still seems like it should be higher to be considered the “core diet”. Almost all of the individual pest species have a wPOO of less than 1% abundance across all sites….

**Answer:** We appreciate the reviewer’s critical perspective on the threshold for "core diet" and the interpretation of wPOO (weighted Percentage of Occurrence) values. However, while we agree that a 5% frequency appears low in absolute terms, this reflects the high taxonomic diversity and "long-tail" distribution typical of generalist insectivore diets captured via DNA metabarcoding. In these highly diverse dietary profiles, in insectivorous bats even the most "heavily consumed" species rarely reach high double-digit percentages because the total diet is spread across hundreds of prey taxa.

We must take into account that our diet study covers what bats ate during their active period (for many months), whereas most pest outbreaks last only a few weeks. Besides, such outbreaks are usually constrained to specific habitats or land-use types within the available foraging grounds for bats. In other words, in heterogeneous landscapes, even during outbreaks, each pest species will constitute only a fraction of prey availability for bats, for only a few weeks. Consequently, even in the most favourable consumption scenario, we shouldn't expect them to reach much higher wPOO values.

To clarify this nuance, we have revised the text. Instead of implying these pests dominate the diet in volume, we now clarify that their importance lies in their status as the most frequently and consistently detected pest taxa relative to the many other pests that appear only as "accidental" or "trace" detections (wPOO < 0.1%).

Besides, we have adjusted the wording on Line 287 (in the previous version) to replace "heavily consumed" with "consistently detected" to more accurately reflect the data. (lines 342-343 in the revised version)

**Line 293:** Again, “manage pest populations effectively” is a bit strong.

**Answer:** We agree with the reviewer that "manage pest populations effectively" implies a level of population control that was not directly measured in this study. Our results demonstrate the consumption of a wide variety of agricultural and forest pests, but do not quantify the resulting reduction in pest density. Accordingly, we have revised this sentence to reflect more accurately the provision of ecosystem services and the potential for biological suppression based on the dietary breadth of these generalist species. (line 365)

**Line 324:** You didn’t prove that there R.hi had a significant impact on pest species- just that they ate a lot of them. Their consumption may have been a drop in the bucket of *O. sacchari* abundance levels- we just don’t know.

**Answer:** OK, we agree that we cannot definitively state that this predation resulted in a numerical "impact" or reduction in total pest abundance. We have therefore revised the text to clarify that *R. hipposideros* exhibits a high frequency of predation on *O. sacchari* during its peak occurrence, representing a concentrated predatory pressure rather than a proven population-level impact. (line 401)

**Figure 1.** I think some of these site names are mixed up. Shouldn’t the labels read as Matxinbenta (MA) and Balmeseda (BA) instead of the species names? Or if that is correct, I don’t understand why and as such this should be added to the figure caption.

**Answer:** We agree with the reviewer and apologise for the error. We had inadvertently used species-based acronyms for these locations. We have corrected Figure 1 so that the labels now correctly reflect the site names: BA for Balmaseda and MA for Matxinbenta.
