# Supplementary material for: Pest consumption by common bats in diverse landscapes
Source: PLoS One. 2026 Jun 17;21(6):e0347853. doi: 10.1371/journal.pone.0347853 (PMC13274907; doi:10.1371/journal.pone.0347853)
Supplement: Peer review history

# Peer review: Decision Letter 0

**DOI:** 10.1371/journal.pone.0347853.r001

**Authors:** Mark Davis

5 Nov 2025

PONE-D-25-25129

Pest consumption by common bats in diverse landscapes

PLOS ONE

Dear Dr. Aldasoro,

Thank you for submitting your manuscript to PLOS ONE. After careful consideration, we feel that it has merit but does not fully meet PLOS ONE’s publication criteria as it currently stands. Therefore, we invite you to submit a revised version of the manuscript that addresses the points raised during the review process.

Two reviewers provided extensive constructive feedback on this manuscript, with both suggesting that a considerable re-write is necessary to improve transparency and reduce vagueness. More critically, a number of assertions are made and inferences are drawn that lack support from presented data and/or the broad body of literature on this topic.

Please submit your revised manuscript by Dec 20 2025 11:59PM. If you will need more time than this to complete your revisions, please reply to this message or contact the journal office at plosone@plos.org. When you're ready to submit your revision, log on to https://www.editorialmanager.com/pone/ and select the 'Submissions Needing Revision' folder to locate your manuscript file.

Please include the following items when submitting your revised manuscript:

- A rebuttal letter that responds to each point raised by the academic editor and reviewer(s). You should upload this letter as a separate file labeled 'Response to Reviewers'.
- A marked-up copy of your manuscript that highlights changes made to the original version. You should upload this as a separate file labeled 'Revised Manuscript with Track Changes'.
- An unmarked version of your revised paper without tracked changes. You should upload this as a separate file labeled 'Manuscript'.

If you would like to make changes to your financial disclosure, please include your updated statement in your cover letter. Guidelines for resubmitting your figure files are available below the reviewer comments at the end of this letter.

If applicable, we recommend that you deposit your laboratory protocols in protocols.io to enhance the reproducibility of your results. Protocols.io assigns your protocol its own identifier (DOI) so that it can be cited independently in the future. For instructions see: https://journals.plos.org/plosone/s/submission-guidelines#loc-laboratory-protocols. Additionally, PLOS ONE offers an option for publishing peer-reviewed Lab Protocol articles, which describe protocols hosted on protocols.io. Read more information on sharing protocols at https://plos.org/protocols?utm_medium=editorial-email&utm_source=authorletters&utm_campaign=protocols.

We look forward to receiving your revised manuscript.

Kind regards,

Mark A. Davis, Ph.D.

Academic Editor

PLOS ONE

Journal requirements:

When submitting your revision, we need you to address these additional requirements.

1. Please ensure that your manuscript meets PLOS ONE's style requirements, including those for file naming. The PLOS ONE style templates can be found at

https://journals.plos.org/plosone/s/file?id=wjVg/PLOSOne_formatting_sample_main_body.pdf and

https://journals.plos.org/plosone/s/file?id=ba62/PLOSOne_formatting_sample_title_authors_affiliations.pdf

2. Thank you for stating the following financial disclosure:

“This research was funded by grant PID2019-108123GB-I00 funded by MICIU/AEI/10.13039/501100011033 and the European Union, and by the Basque Government (Research grant IT1571-22) and its Biodiversity Directorate (Expedient No 049‐2022‐42).”

Please state what role the funders took in the study.  If the funders had no role, please state: "The funders had no role in study design, data collection and analysis, decision to publish, or preparation of the manuscript."

If this statement is not correct you must amend it as needed.

Please include this amended Role of Funder statement in your cover letter; we will change the online submission form on your behalf.

3. Thank you for stating the following in the Funding Section of your manuscript:

“This research was funded by grant PID2019-108123GB-I00 funded by MICIU/AEI/10.13039/501100011033 and the European Union, and by the Basque Government (Research grant IT1571-22) and its Biodiversity Directorate (Expedient No 049‐2022‐42).”

We note that you have provided funding information that is currently declared in your Funding Statement. However, funding information should not appear in the Acknowledgments section or other areas of your manuscript. We will only publish funding information present in the Funding Statement section of the online submission form.

Please remove any funding-related text from the manuscript and let us know how you would like to update your Funding Statement. Currently, your Funding Statement reads as follows:

“This research was funded by grant PID2019-108123GB-I00 funded by MICIU/AEI/10.13039/501100011033 and the European Union, and by the Basque Government (Research grant IT1571-22) and its Biodiversity Directorate (Expedient No 049‐2022‐42).”

Please include your amended statements within your cover letter; we will change the online submission form on your behalf.

4. When completing the data availability statement of the submission form, you indicated that you will make your data available on acceptance. We strongly recommend all authors decide on a data sharing plan before acceptance, as the process can be lengthy and hold up publication timelines. Please note that, though access restrictions are acceptable now, your entire data will need to be made freely accessible if your manuscript is accepted for publication. This policy applies to all data except where public deposition would breach compliance with the protocol approved by your research ethics board. If you are unable to adhere to our open data policy, please kindly revise your statement to explain your reasoning and we will seek the editor's input on an exemption. Please be assured that, once you have provided your new statement, the assessment of your exemption will not hold up the peer review process.

If the reviewer comments include a recommendation to cite specific previously published works, please review and evaluate these publications to determine whether they are relevant and should be cited. There is no requirement to cite these works unless the editor has indicated otherwise.

Additional Editor Comments:

Two reviewers found that this research has merit, but both identified a number of areas that must be addressed before this manuscript can be considered for publication. Reviewer 1 identified a number of areas where the manuscript requires revisions to improve clarity and reduce vagueness. Reviewer 2 identifies a number of assertions made and inferences drawn that have neither support from the data, nor evidence from published literature. These reviews suggest extensive revisions are necessary.

[Note: HTML markup is below. Please do not edit.]

Reviewers' comments:

Reviewer's Responses to Questions

**Comments to the Author**

1. Is the manuscript technically sound, and do the data support the conclusions?

The manuscript must describe a technically sound piece of scientific research with data that supports the conclusions. Experiments must have been conducted rigorously, with appropriate controls, replication, and sample sizes. The conclusions must be drawn appropriately based on the data presented.

Reviewer #1: Partly

Reviewer #2: Partly

**********

2. Has the statistical analysis been performed appropriately and rigorously?

Reviewer #1: Yes

Reviewer #2: Yes

**********

3. Have the authors made all data underlying the findings in their manuscript fully available?

The PLOS Data policy requires authors to make all data underlying the findings described in their manuscript fully available without restriction, with rare exception (please refer to the Data Availability Statement in the manuscript PDF file). The data should be provided as part of the manuscript or its supporting information, or deposited to a public repository. For example, in addition to summary statistics, the data points behind means, medians and variance measures should be available. If there are restrictions on publicly sharing data—e.g. participant privacy or use of data from a third party—those must be specified.

Reviewer #1: Yes

Reviewer #2: No

**********

4. Is the manuscript presented in an intelligible fashion and written in standard English?

PLOS ONE does not copyedit accepted manuscripts, so the language in submitted articles must be clear, correct, and unambiguous. Any typographical or grammatical errors should be corrected at revision, so please note any specific errors here.

Reviewer #1: Yes

Reviewer #2: Yes

**********

5. Review Comments to the Author

Please use the space provided to explain your answers to the questions above. You may also include additional comments for the author, including concerns about dual publication, research ethics, or publication ethics. (Please upload your review as an attachment if it exceeds 20,000 characters)

Reviewer #1: Journal: PLOS ONE

Manuscript ID: PONE-D-25-25129

Pest consumption by common bats in diverse landscapes

This manuscript explores how bats provide essential ecosystem services in the form of insect pest consumption. Using Rhinolophus bats as focal species, the authors used insect DNA found in fecal samples to estimate the impact bats were having on insect pest species in the region.

Main Comments:

• The introduction introduces the material well, but there are parts where the language is vague and there are not good connections between paragraphs. I recommend spending more time linking the concepts to why the focal species is a good model a bit more. Additionally, spend more time explaining on how insects are considered pest species in the region and connecting it to your focal species.

• Overall there is excellent information herein, but there is a lot of vague language used and there needs to be more time spent connecting concepts together.

• There are some changes needed for the tables and figures to make them cleaner and easier to understand. Always remember that the reader should be able to just read the captions and know what the figure is about without ever seeing it.

• Because the reader may be unfamiliar with the region, I recommend including a map with where the site are located/where samples were collected.

• The abstract states that the study is during the breeding season, but does not explain well more of the life history of the focal species. The study covers 7 months, which may encompass the breeding season and then some. More care should be given introducing the focal species and what they are a good model for this study (which I do think they are).

Specific suggestions and remarks

Introduction

37-38: Reduce sentence length “…feed on a wide variety of arthropods, including many pest species such as …”

40: Vague language; “This makes bats vital…”

42: Bats can offer; leave the potential open ended so that it gives the reader the sense that your work is adding to the collection of work

46: Plasticity of what? Be more specific and then give examples of those.

51-52: Avoid vague words such as “this”. Consider rephrasing “Because of bats ability to adjust their diets and foraging habitat, they are highly effective pest suppressors…”

60-61: Inappropriate to call other studies “run of the mill”. It gives the wrong impression about their value

60-67: I would consider reframing this paragraph to focus on how fecal DNA is a great tool to understand bat diets, and thus the pests they consume.

68-79: These two paragraphs do not connect to one another well. Maybe spend time on why it is important to use abundant and widely distributed species can help answer the question, and then go into your focal species as a model.

Methods

82: Be more specific as to where this study is occurring. It may be worth including a map of the region, and where samples were collected.

90-99: Inconsistent with time periods. Some state the week collection occurred, others just the month/day. Be consistent.

100-105: You introduce “OUT”s here, but do not explain what it is. It is explained later on, but currently the reader does not know what OTUs are. I suggest either expanding this section, or moving it further down to be aligned with the description of OTU.

145: If it is unpublished, how are the data accessible?

151: If focusing on these uncertainties, spend more time in the intro about the specifics.

156-170: It says there is a formula, but I do not see one written out.

Results

174: Were the samples pooled? Or were they tested individually? Is this proportion just the samples that tested for any pest species? What is the proportion of pest species within the positive samples?

192-193: Linked how? Be more specific.

193-195: Species of bat or of pest insect? Be more specific and how they are linked to the plant species.

230-232: How do we, the reader, know that is the usual time pest species emerge? What does “typical emergence pattern” mean? Is it species specific?

243-245: State earlier species general differences, such as their sizes.

251-252: Why do they have lower energy costs? If this is an assumption because of size, I suggest removal of the sentence. If it is supported in the literature, then cite the sources.

Discussion

257: That some species of horseshoe bats.

257-258: Get rid of “because of their remarkable adaptability”. Suggest it later in the discussion if appropriate.

260: Remove “in this study”

261-263: Combine sentences; “…resulting in few total occurrences, while other insect species are heavily consumed and form what is known as “core diet”..”

263: “These” is vague; “The insects comprising the core species…”

265-266: Explain more the adaptability you document ahead of the ending sentence such as “Because we documented bats doing x,y,z, we argue that they have an adaptable diet and thus…”

270-271: This is not really what your study looked at, but your study does suggest it. Propose the ideas as potential hypotheses, not as fact.

278-281: This sentence seems out of place compared to the topic sentence.

299-305: I think this paragraph, while interesting, is not necessary and could be removed or incorporated elsewhere.

Tables and Figure Captions

Table 1:

Table 2: Add more to the table caption including writing out the full site names and species names.

Table 3: Use decimal points rather than commas.

Table 4: A bit confusing; the total diet is less than individual sites. Explain more in the caption so that the reported values make more sense.

Figure 1: Explain how sampling week is in relation to the week of the year somewhere.

Figure 2: Spell out full bat species name. The metric is needed (%?) for the wPOO. What are the units?

Figure 3: Units needed on (a); Kg of what? Put more on axis label

Figure 4: Is this for both species or just one? State it in the figure caption.

Figure 5: Spell out full bat species name. Add units on axis.

Reviewer #2: Peer Review: “Pest consumption by common bats in diverse landscapes” by Aldasoro

The manuscript, “Pest consumption by common bats in diverse landscapes”, details an important narrative of the ecosystem service that bats provide as pest consumers. Quantifying the abundance and diversity of pests that bats consume in varying agricultural settings throughout a season plays an important role in promoting bat conservation and overall ecological health. This manuscript supports this foundational knowledge by studying two common & abundant species in Spain and describing their pest consumption throughout the season. While the manuscript makes several claims without citations or data to support them, with further citations and specifications, this manuscript will benefit the body of literature well.

I think my biggest issue with this manuscript is that it makes the claim that the seasonality in consumption of different pests follows the pulses in pest abundance associated with hatches. However, there are no citations or data that support the seasonality of pulses in pest abundance. Without supporting data to show that the peak in species-specific pest abundance mirrors the peak in species-specific consumption, I don’t think you can make this claim. I believe that this is a highly-probable theory, but the diet data that you are analyzing doesn’t support this claim until you add additional data about seasonality of the pest abundance.

Additionally, you also make the claim that lower pest abundance comes from sites that have more diverse and natural surroundings, however you don’t provide any data to back this claim up. This seems like an easily quantifiable fix with land use imagery to quantity the amount of natural area to anthropisation around each site, but without the data to support this- its an unsubstantiated claim in the paper. Detailed in the comments below are more instances where more specifics are needed or claims are quite broad.

Specific Edits:

Line 27: “they can go one step beyond human capabilities” – Please be specific about what you mean here.

Line 29: “several tons of insects” – Give a range of values that you have found here.

Line 50: “frequently consume insects from outside their hunting grounds” – I would say that many bats do not have specific hunting grounds, but forage opportunistically as you mention. I think just rewording this phrase would be best.

Line 56: “The larger the population, the greater the collective consumption of pests” – This seems like an assumption to me and I could think of many ecological scenarios where abundance of bats does not correlate with pests consumed. I would rephrase this to accommodate some of this uncertainty.

Line 60: “Run-of-the-mill” – This seems a little derogative to the studies mentioned- I would choose a different descriptor.

Line 65: What method have these studies used to estimate pest consumption? You refer back to this later, so I think it would be helpful to be specific here.

Line 77-79: I think a clearer description of research question, study objective, and study importance would be beneficial here. The last sentence just feels tacked on without really incorporating it into the study importance. Just rephrasing this would create a much stronger introduction

Line 83: Double check your species names here- I think you can start using abbreviations for both species. This happens several times throughout the paper including with the pest species, but I am not going to list them all.

Table 1: Population sizes in colonies vary quite a bit over the course of the year as juveniles are born. Are these populations sizes pre- or post- birth pulse in abundance?

Line 97: “Up to 24 samples of 4-6 pellets” - How did you decide on how many samples per session? How did you did you decide on which pellets to pick up? Randomly? Off the top? Largest? Most whole pellets?

Line 101: How many samples were considered contaminated from the 413 and 377 samples?

Line 102: Here you say you are analyzing them based on sampling month, but some of your figures are divided up by sampling week? Is that just for R. ferrumquinum? I’m a little confused about this part.

Line 142: How did you decide what was potential prey and what wasn’t?

Line 154: I would love to see a map of site locations and this general region in Spain- I think this would help delinate some of the later population size estimates.

Line 156: You outline your variables here, but I never see an actual formula about how you calculate these together?

Line 160: It would be good to know the where the 30% comes from in these studies as compared to the 70% in c1. Why such a big difference between the two? Are the studies for c2 also during pregnancy and lactation? Or males? If you just chose the min and max values from the literature, I would just state it as such.

Line 163: “Basque Country of Aihzartza (2001)” – Reword: it sounds like there is a country in Basque named Aihzarta?

Table 3: I understand that you are using wPOO here, but it feels a little nebulous to me. If the max that a wPOO can be is 100% (where they only eat one thing), then 8.5% of insects that R. hipposideros eats is a pest seems rather low?

Figure 2a: I would reduce the y-axis range down to 1500 wPOO. Since there is only one main high point in that range, it would be more easier to read the smaller values.

Line 232: I think this is a key point in your paper, but it would be a lot stronger to have data to back this up. Does the Integrated Pest Management Guides of the Spanish Government have temporal data to cite for an abundance comparison with the peak consumption? Otherwise, you are stating this with no citation or evidence to back the claim up.

Line 245 & 247: You now use the common name here- I would stick to either Latin name or common name.

Line 248: I think the map part may help a bit here, but how far of a spatial scale would the 79.28kg eaten be covering? An estimate of the home range of the colonies would be beneficial here. Would you expect this estimate to vary between colonies? Are the ranges in colonies overlapping?

Line 254: This is the first time that you mention anthropisation in the paper. How did you calculate this? Which sites are higher or lower in terms of anthropisaion?

Line 257: Based on what you just presented, it seems like they eat more than “several” agroforestry pests?

Line 264 &267-268: Again, I think your work would be a lot stronger if you had data to compare the peak outbreaks of pests with the peaks in consumption and showed that bats are moving from one pest to the next and are truly following the varying peaks in abundance. Right now, you just show us that consumptions changes throughout the season without proving to us that it follows peak abundance. I get that you may not have data to prove localized abundance, but even literature supporting the timing of these peaks would be strengthen your work.

Line 269: “typical general” – I think you only need one of these words here.

Line 274: Again, a map to see these rivers would be great. I also want to emphasize again that you haven’t provided us any context or data to differentiate between sites in the amount of “diverse natural habitat” nearby. Maybe in Table 1- you could put a percentage of natural habitat vs anthropogenic habitat? Its hard to know if you consider a pine plantation as natural or anthropogenic otherwise or what the difference is between Mediterranean bush vs shrubland?

Line 279-281: “bats’ impact can exceed their foraging areas”- How do we know they aren’t foraging down there? Are the insects moving to the bats or vice versa? Or do the rice pests utilize other plants as well?

Line 297: “horseshoe bats are among the most important bat species for pest suppression” – This seems like a bit of a stretch to me for the subject of this paper. How do we know that other species do not eat higher rates of pests? Are they the biggest bat in the region in which case, you could rephrase this comment? Maybe caveat it with “Given that horseshoe bats are one of the most common and can be one of larger-bodied bats in the region, …”

Line 314: “one step beyond human capabilities” – Again, I feel like this phrasing is a little weird. I would reword since I can see what you are saying here.

Line 326: I also think a line in here about education on the importance of bats and their ecosystem services within the public would also be important for maintaining bat populations and positive bat sentiments.

**********

6. PLOS authors have the option to publish the peer review history of their article (what does this mean?). If published, this will include your full peer review and any attached files.

If you choose “no”, your identity will remain anonymous but your review may still be made public.

**Do you want your identity to be public for this peer review?** For information about this choice, including consent withdrawal, please see our Privacy Policy.

Reviewer #1: No

Reviewer #2: No

**********

[NOTE: If reviewer comments were submitted as an attachment file, they will be attached to this email and accessible via the submission site. Please log into your account, locate the manuscript record, and check for the action link "View Attachments". If this link does not appear, there are no attachment files.]

To ensure your figures meet our technical requirements, please review our figure guidelines: https://journals.plos.org/plosone/s/figures

You may also use PLOS’s free figure tool, NAAS, to help you prepare publication quality figures: https://journals.plos.org/plosone/s/figures#loc-tools-for-figure-preparation.

NAAS will assess whether your figures meet our technical requirements by comparing each figure against our figure specifications.

# Author comment: Author response to Decision Letter 1

**DOI:** 10.1371/journal.pone.0347853.r002

9 Jan 2026

Reviewer #1: Journal: PLOS ONE

This manuscript explores how bats provide essential ecosystem services in the form of insect pest consumption. Using Rhinolophus bats as focal species, the authors used insect DNA found in fecal samples to estimate the impact bats were having on insect pest species in the region.

Main Comments:

• The introduction introduces the material well, but there are parts where the language is vague and there are not good connections between paragraphs. I recommend spending more time linking the concepts to why the focal species is a good model a bit more. Additionally, spend more time explaining on how insects are considered pest species in the region and connecting it to your focal species.

• Overall there is excellent information herein, but there is a lot of vague language used and there needs to be more time spent connecting concepts together.

• There are some changes needed for the tables and figures to make them cleaner and easier to understand. Always remember that the reader should be able to just read the captions and know what the figure is about without ever seeing it.

• Because the reader may be unfamiliar with the region, I recommend including a map with where the site are located/where samples were collected.

• The abstract states that the study is during the breeding season, but does not explain well more of the life history of the focal species. The study covers 7 months, which may encompass the breeding season and then some. More care should be given introducing the focal species and what they are a good model for this study (which I do think they are).

Dear Reviewer,

We sincerely thank you for your thorough and constructive evaluation of our manuscript. Your comments have greatly contributed to improving the clarity, structure, and overall quality of our work. We have carefully addressed each of your points and revised the manuscript accordingly. Below, we provide detailed responses to all suggestions, indicating where modifications were made in the revised version.

We substantially revised the Introduction to improve conceptual flow, clarify the framing, and reinforce the relevance of the focal species. We expanded the explanation of why horseshoe bats represent an appropriate model for assessing bat-mediated pest suppression.

We revised the text extensively to eliminate vague wording and strengthen conceptual connections across sections. Many sentences and paragraphs were rephrased for precision and clarity, following your suggestions.

We revised all figure and table captions to be more detailed and self-explanatory and applied formatting adjustments to the tables and axis labels as recommended. Explanations of units and terminology were added where needed.

In addition, we have added a new map illustrating the locations of all sampled colonies (new Figure 1).

Specific suggestions and remarks

Introduction

37-38: Reduce sentence length “…feed on a wide variety of arthropods, including many pest species such as …”

Answer: OK, we rephrased the sentence. (lines 36-37 of the revised manuscript)

40: Vague language; “This makes bats vital…”

Answer: OK, we rephrased the sentence. (lines 38-39)

42: Bats can offer; leave the potential open ended so that it gives the reader the sense that your work is adding to the collection of work

Answer: OK, we included “can” in the sentence (line 40)

46: Plasticity of what? Be more specific and then give examples of those.

Answer: OK, we rephrased the sentence. (lines 43-45)

51-52: Avoid vague words such as “this”. Consider rephrasing “Because of bats ability to adjust their diets and foraging habitat, they are highly effective pest suppressors…”

Answer: OK, we rephrased the sentence. (lines 49-51)

60-61: Inappropriate to call other studies “run of the mill”. It gives the wrong impression about their value

Answer: We rephrased the paragraph and removed this phrase (next point). (lines 52-65)

60-67: I would consider reframing this paragraph to focus on how fecal DNA is a great tool to understand bat diets, and thus the pests they consume.

Answer: OK, we changed the paragraph to emphasise the role of metabarcoding. (lines 52-65)

68-79: These two paragraphs do not connect to one another well. Maybe spend time on why it is important to use abundant and widely distributed species can help Answer the question, and then go into your focal species as a model.

Answer: OK, following this suggestion we changed the previous paragraph about the importance of abundant and common species to link both paragraphs. (lines 66-83)

Methods

82: Be more specific as to where this study is occurring. It may be worth including a map of the region, and where samples were collected.

Answer: We included a figure with the location of the sampled colonies. (new Figure 1)

90-99: Inconsistent with time periods. Some state the week collection occurred, others just the month/day. Be consistent.

Answer: OK, we changed the time periods to always include the Julian week and the month (lines 97-103)

100-105: You introduce “OUT”s here, but do not explain what it is. It is explained later on, but currently the reader does not know what OTUs are. I suggest either expanding this section, or moving it further down to be aligned with the description of OTU.

Answer: OK, we added the explanation of “OTU” in the current sentence. (lines 117-118)

145: If it is unpublished, how are the data accessible?

Answer: At the current moment, it has already been published, so the reference “Aldasoro et al., 2025b” has been added. (line 159)

151: If focusing on these uncertainties, spend more time in the intro about the specifics.

Answer: Yes, the new version of the Introduction addresses this issue (lines 52-65).

156-170: It says there is a formula, but I do not see one written out.

Answer: Sorry about that; it looks like the format change caused it to disappear. We added the formula (line 170)

Results

174: Were the samples pooled? Or were they tested individually? Is this proportion just the samples that tested for any pest species? What is the proportion of pest species within the positive samples?

Answer: We included an explanation of sample collection in the ‘Sampling Bat Droppings’ section (lines 106-108). All the samples included in the study were positive for arhropod prey. The reported proportion refers to the fraction of faecal (community) samples that tested positive for any pest species.

192-193: Linked how? Be more specific.

Answer: OK, we rephrased the sentence. (lines 209-212)

193-195: Species of bat or of pest insect? Be more specific and how they are linked to the plant species.

Answer: OK, we rephrased the sentence (lines 210-211)

Since these are pest species of agricultural crops, their link / association indicates that they also pose a threat to these specific cultivars.

230-232: How do we, the reader, know that is the usual time pest species emerge? What does “typical emergence pattern” mean? Is it species specific?

Answer: While the Integrated Pest Management Guides do not provide detailed temporal abundance data, they do indicate the peak seasons for each pest species, which aligns with our observations of peak bat consumption. We have added the citation to the text: (Gil et al. 2014). (lines 47-48). We have also revised the corresponding statements in the manuscript to ensure they accurately reflect the resolution of our data while maintaining alignment with the information available in the literature.

243-245: State earlier species general differences, such as their sizes.

Answer: OK, we included a short sentences stating these differences in the introduction. (lines 74-79)

251-252: Why do they have lower energy costs? If this is an assumption because of size, I suggest removal of the sentence. If it is supported in the literature, then cite the sources.

Answer: No, you are right. To our knowledge there is not any information about that. We rewrote the sentence to make it clearer. (lines 269-271)

Discussion

257: That some species of horseshoe bats.

Answer: OK, we changed the sentence. (line 281)

257-258: Get rid of “because of their remarkable adaptability”. Suggest it later in the discussion if appropriate.

Answer: OK, removed.

260: Remove “in this study”

Answer: OK, removed.

261-263: Combine sentences; “…resulting in few total occurrences, while other insect species are heavily consumed and form what is known as “core diet”.”

Answer: OK, we combined the sentences as suggested (lines 285-286)

263: “These” is vague; “The insects comprising the core species…”

Answer: OK, we changed the sentence as suggested (lines 287)

265-266: Explain more the adaptability you document ahead of the ending sentence such as “Because we documented bats doing x,y,z, we argue that they have an adaptable diet and thus…”

Answer: OK, we included a previous sentence pointing out this adaptability. (lines 289-291)

270-271: This is not really what your study looked at, but your study does suggest it. Propose the ideas as potential hypotheses, not as fact.

Answer: OK, we agree and changed the sentence accordingly. (lines 296-297)

278-281: This sentence seems out of place compared to the topic sentence.

Answer: OK, we agree and changed that sentence to the end of the previous paragraph (lines 297-300)

299-305: I think this paragraph, while interesting, is not necessary and could be removed or incorporated elsewhere.

Answer: Here we disagree. In fact, in our opinion, one of the most interesting findings on the trophic ecology of insectivorous bats during the last years is that bat species belonging to different guilds —and therefore with different foraging adaptations/preferences— tend to converge, opportunistically exploiting the same insect outbreaks (e..g. Garin et al. 2019; Aihartza et al. 2023; Aldasoro et al. 2025a and 2025b...). So, we would keep this paragraph if the editor agrees. (lines 326-334)

Tables and Figure Captions

Table 2: Add more to the table caption including writing out the full site names and species names.

Answer: OK, we added the required information to the table caption. (lines 186-189)

Table 3: Use decimal points rather than commas.

Answer: OK, changed to decimal points. (lines 223-227)

Table 4: A bit confusing; the total diet is less than individual sites. Explain more in the caption so that the reported values make more sense.

Answer: We agree that this can appear confusing. The weighted percentage of occurrence reflects the proportion of occurrences within each site. For individual colonies, a given pest group can represent a relatively high percentage because it is calculated only from that colony’s samples. When all colonies are combined, the total number of samples increases substantially, so the same pest group represents a smaller proportion in the overall dataset. We clarified this in the table caption to make the values easier to interpret (and also in Table 3). (lines 257-260 and 223-227)

Figure 1: Explain how sampling week is in relation to the week of the year somewhere.

Answer: OK, we added month names under the Julian weeks (Now Figure 2)

Figure 2: Spell out full bat species name. The metric is needed (%?) for the wPOO. What are the units?

Answer: OK, we added the full species name to the figure caption and added (%) to the y axis. (Now Figure 3) (lines 216-218)

Figure 3: Units needed on (a); Kg of what? Put more on axis label

Answer: OK, we added (%) to the y axis in (a) and “Pest Kg” to (b). (Now Figure 4)

Figure 4: Is this for both species or just one? State it in the figure caption.

Answer: OK, we added “Rhinolophus ferrumequinum” to the caption. (Now Figure 5) (lines 244-246)

Figure 5: Spell out full bat species name. Add units on axis.

Answer: OK, added the full species name to the figure caption and added (%) to the y-axis. (Now Figure 6) (lines 254-256)

Reviewer #2:

The manuscript, “Pest consumption by common bats in diverse landscapes”, details an important narrative of the ecosystem service that bats provide as pest consumers. Quantifying the abundance and diversity of pests that bats consume in varying agricultural settings throughout a season plays an important role in promoting bat conservation and overall ecological health. This manuscript supports this foundational knowledge by studying two common & abundant species in Spain and describing their pest consumption throughout the season. While the manuscript makes several claims without citations or data to support them, with further citations and specifications, this manuscript will benefit the body of literature well.

I think my biggest issue with this manuscript is that it makes the claim that the seasonality in consumption of different pests follows the pulses in pest abundance associated with hatches. However, there are no citations or data that support the seasonality of pulses in pest abundance. Without supporting data to show that the peak in species-specific pest abundance mirrors the peak in species-specific consumption, I don’t think you can make this claim. I believe that this is a highly-probable theory, but the diet data that you are analyzing doesn’t support this claim until you add additional data about seasonality of the pest abundance.

Additionally, you also make the claim that lower pest abundance comes from sites that have more diverse and natural surroundings, however you don’t provide any data to back this claim up. This seems like an easily quantifiable fix with land use imagery to quantity the amount of natural area to anthropisation around each site, but without the data to support this- its an unsubstantiated claim in the paper. Detailed in the comments below are more instances where more specifics are needed or claims are quite broad.

Dear Reviewer,

We sincerely thank you for your thorough and constructive evaluation of our manuscript. Your comments have greatly contributed to improving the clarity, rigor, and overall quality of our work. We have carefully addressed each of your points and revised the manuscript accordingly. Below, we provide detailed responses to your specific comments, indicating where modifications were made in the revised version.

Regarding your primary concern about the seasonality of pest consumption, although fine-scale temporal abundance data for pest species are not available, the Integrated Pest Management Guides provide information on the general peak seasons for each pest species. These documented seasonal peaks align with the periods in which we observed elevated bat consumption (Gil et al., 2014). The manuscript has been revised to clarify that our findings are consistent with these documented seasonal trends while accurately reflecting the resolution of our data.

Concerning landscape effects on pest abundance, this study builds on prior work (Aldasoro et al., 2025a, 2025b), which quantified land use around each colony. Relevant information has now been added to Table 1, detailing the proportion of natural versus anthropogenic habitats, thereby providing context for observed differences in pest abundance across sites.

Specific Edits:

Line 27: “they can go one step beyond human capabilities” – Please be specific about what you mean here.

Answer: OK, we rewrote the sentence to be clearer. (lines 24-26 of the revised manuscript)

Line 29: “several tons of insects” – Give a range of values that you have found here.

Answer: OK, we changed the sentence (line 28)

Line 50: “frequently consume insects from outside their hunting grounds” – I would say that many bats do not have specific hunting grounds, but forage opportunistically as you mention. I think just rewording this phrase would be best.

Answer: OK, we rephrased the sentence to include the opportunistic option. Nevertheless, Arrizabalaga et al. (2015) reported that the source-habitat of many of the main prey consumed by Mediterranean horseshoes didn’t fit their preferred foraging habitats, thoroughly studied by radio-tracking in the very same study area. (lines 46-49)

Line 56: “The larger the population, the greater the collective consumption of pests” – This seems like an assumption to me and I could think of many ecological scenarios where ab

Attachment Submitted filename: Response to reviewers.docx

# Peer review: Decision Letter 1

**DOI:** 10.1371/journal.pone.0347853.r003

**Authors:** Mark Davis

4 Mar 2026

PONE-D-25-25129R1

Pest consumption by common bats in diverse landscapes

PLOS One

Dear Dr. Aldasoro,

Thank you for submitting your manuscript to PLOS ONE. After careful consideration, we feel that it has merit but does not fully meet PLOS ONE’s publication criteria as it currently stands. Therefore, we invite you to submit a revised version of the manuscript that addresses the points raised during the review process.

Overall, this manuscript is vastly improved from the original version, and the authors are to be commended for engaging with the reviewers to improve their work. There remain a few outstanding details that Reviewers 1 & 2 identify, and, overall, a careful proofread to address errors in spelling/grammar/syntax/punctuation. Once these issues are resolved, the manuscript will be acceptable for publication in PLoS ONE.

Please submit your revised manuscript by Apr 18 2026 11:59PM. If you will need more time than this to complete your revisions, please reply to this message or contact the journal office at plosone@plos.org. When you're ready to submit your revision, log on to https://www.editorialmanager.com/pone/ and select the 'Submissions Needing Revision' folder to locate your manuscript file.

Please include the following items when submitting your revised manuscript:

- A letter that responds to each point raised by the academic editor and reviewer(s). You should upload this letter as a separate file labeled 'Response to Reviewers'.
- A marked-up copy of your manuscript that highlights changes made to the original version. You should upload this as a separate file labeled 'Revised Manuscript with Track Changes'.
- An unmarked version of your revised paper without tracked changes. You should upload this as a separate file labeled 'Manuscript'.

-->If you would like to make changes to your financial disclosure, please include your updated statement in your cover letter. Guidelines for resubmitting your figure files are available below the reviewer comments at the end of this letter.

If applicable, we recommend that you deposit your laboratory protocols in protocols.io to enhance the reproducibility of your results. Protocols.io assigns your protocol its own identifier (DOI) so that it can be cited independently in the future. For instructions see: https://journals.plos.org/plosone/s/submission-guidelines#loc-laboratory-protocols. Additionally, PLOS ONE offers an option for publishing peer-reviewed Lab Protocol articles, which describe protocols hosted on protocols.io. Read more information on sharing protocols at https://plos.org/protocols?utm_medium=editorial-email&utm_source=authorletters&utm_campaign=protocols.

We look forward to receiving your revised manuscript.

Kind regards,

Mark A. Davis, Ph.D.

Academic Editor

PLOS One

Journal Requirements:

If the reviewer comments include a recommendation to cite specific previously published works, please review and evaluate these publications to determine whether they are relevant and should be cited. There is no requirement to cite these works unless the editor has indicated otherwise.

Please review your reference list to ensure that it is complete and correct. If you have cited papers that have been retracted, please include the rationale for doing so in the manuscript text, or remove these references and replace them with relevant current references. Any changes to the reference list should be mentioned in the rebuttal letter that accompanies your revised manuscript. If you need to cite a retracted article, indicate the article’s retracted status in the References list and also include a citation and full reference for the retraction notice.

Additional Editor Comments:

Graciously, the original two reviewers agreed to review this resubmission. The reviewers agree and I concur that the manuscript has vastly improved since the original version, however their recommendations were split. Reviewer 1 identifies a few remaining issues that need to be address, but argues for minor revisions. Reviewer 2 identifies several areas where additional nuance and context is required, but argues to accept. Both reviewers note that the manuscript, overall, needs a careful revision to address errors in grammar/spelling/syntax/punctuation. Once these minor revisions are complete, this manuscript should be ready for publication in PLoS one.

[Note: HTML markup is below. Please do not edit.]

Reviewers' comments:

Reviewer's Responses to Questions

**Comments to the Author**

1. If the authors have adequately addressed your comments raised in a previous round of review and you feel that this manuscript is now acceptable for publication, you may indicate that here to bypass the “Comments to the Author” section, enter your conflict of interest statement in the “Confidential to Editor” section, and submit your "Accept" recommendation.

Reviewer #1: All comments have been addressed

Reviewer #2: (No Response)

**********

2. Is the manuscript technically sound, and do the data support the conclusions?

The manuscript must describe a technically sound piece of scientific research with data that supports the conclusions. Experiments must have been conducted rigorously, with appropriate controls, replication, and sample sizes. The conclusions must be drawn appropriately based on the data presented.

Reviewer #1: Yes

Reviewer #2: Partly

**********

3. Has the statistical analysis been performed appropriately and rigorously?

Reviewer #1: Yes

Reviewer #2: Yes

**********

4. Have the authors made all data underlying the findings in their manuscript fully available?

The PLOS Data policy requires authors to make all data underlying the findings described in their manuscript fully available without restriction, with rare exception (please refer to the Data Availability Statement in the manuscript PDF file). The data should be provided as part of the manuscript or its supporting information, or deposited to a public repository. For example, in addition to summary statistics, the data points behind means, medians and variance measures should be available. If there are restrictions on publicly sharing data—e.g. participant privacy or use of data from a third party—those must be specified.

Reviewer #1: Yes

Reviewer #2: Yes

**********

5. Is the manuscript presented in an intelligible fashion and written in standard English?

PLOS ONE does not copyedit accepted manuscripts, so the language in submitted articles must be clear, correct, and unambiguous. Any typographical or grammatical errors should be corrected at revision, so please note any specific errors here.

Reviewer #1: Yes

Reviewer #2: Yes

**********

6. Review Comments to the Author

Please use the space provided to explain your answers to the questions above. You may also include additional comments for the author, including concerns about dual publication, research ethics, or publication ethics. (Please upload your review as an attachment if it exceeds 20,000 characters)

Reviewer #1: Journal: PLOS ONE

Manuscript ID: PONE-D-25-25129

Second Review

Pest consumption by common bats in diverse landscapes

This manuscript explores how bats provide essential ecosystem services in the form of insect pest consumption. Using Rhinolophus bats as focal species, the authors used insect DNA found in fecal samples to estimate the impact bats were having on insect pest species in the region.

Main Comments:

• The authors did a diligent job on addressing the comments and critiques from the first review. The manuscript has greatly improved, and I only have a few small recommendations. The manuscript is greatly improved and provides much better context for the work and findings.

Specific suggestions and remarks

Introduction

75-76: Consider separating into two paragraphs with the species info in the next

Methods

88-89: Be more specific as to where this study is occurring; state the country.

91: Expand the figure description. What is the inset map? What is it focusing in on?

122-128: Move the ethics statement up further

Reviewer #2: Thank you for your revision & thoughtful responses to reviewer comments. I believe that your manuscript is stronger because of these revisions. There are still some grammatical and spelling errors throughout the manuscript. Mainly this is a topic with a lot of nuances and I have listed out a couple spots below where I feel the nuance may still need to be addressed.

Line 171: I would organize these descriptions based on the order they appear in your equation. Also your equation uses ‘pop’ and your description uses ‘pop size’ – these should be identical. I would also point out that you are using a population estimate from 2001 which is 25 years ago. Do you expect these population estimates to have changed at all? You also report that ‘other studies’ report similar population sizes between the two, but don’t cite these studies.

Line 287: I still feel a little conflicted about something that occurs in 5% of the total samples across all colonies being considered as “heavily consumed”. I understand that you have a citation to support this, but to me it still seems like it should be higher to be considered the “core diet”. Almost all of the individual pest species have a wPOO of less than 1% abundance across all sites….

Line 293: Again, “manage pest populations effectively” is a bit strong.

Line 324: You didn’t prove that there R.hi had a significant impact on pest species- just that they ate a lot of them. Their consumption may have been a drop in the bucket of O. sacchari abundance levels- we just don’t know.

Figure 1. I think some of these site names are mixed up. Shouldn’t the labels read as Matxinbenta (MA) and Balmeseda (BA) instead of the species names? Or if that is correct, I don’t understand why and as such this should be added to the figure caption.

**********

7. PLOS authors have the option to publish the peer review history of their article (what does this mean?). If published, this will include your full peer review and any attached files.

If you choose “no”, your identity will remain anonymous but your review may still be made public.

**Do you want your identity to be public for this peer review?** For information about this choice, including consent withdrawal, please see our Privacy Policy.

Reviewer #1: No

Reviewer #2: No

**********

[NOTE: If reviewer comments were submitted as an attachment file, they will be attached to this email and accessible via the submission site. Please log into your account, locate the manuscript record, and check for the action link "View Attachments". If this link does not appear, there are no attachment files.]

To ensure your figures meet our technical requirements, please review our figure guidelines: https://journals.plos.org/plosone/s/figures

You may also use PLOS’s free figure tool, NAAS, to help you prepare publication quality figures: https://journals.plos.org/plosone/s/figures#loc-tools-for-figure-preparation.

NAAS will assess whether your figures meet our technical requirements by comparing each figure against our figure specifications.

Attachment Submitted filename: SecondReview_PONE-D-25-25129.pdf

# Author comment: Author response to Decision Letter 2

**DOI:** 10.1371/journal.pone.0347853.r004

24 Mar 2026

Dear Dr Davis,

We thank you and the reviewers for the thoughtful evaluation of our revised manuscript. We are pleased to note the positive feedback regarding the improvements made to the paper. We have addressed the remaining points regarding geographic clarity, terminology, and ecological nuance.

Below, we provide a point-by-point response to the comments provided by the Editor and the Reviewers.

Reviewer #1: Journal: PLOS ONE

This manuscript explores how bats provide essential ecosystem services in the form of insect pest consumption. Using Rhinolophus bats as focal species, the authors used insect DNA found in fecal samples to estimate the impact bats were having on insect pest species in the region.

Main Comments:

• The authors did a diligent job on addressing the comments and critiques from the first review. The manuscript has greatly improved, and I only have a few small recommendations. The manuscript is greatly improved and provides much better context for the work and findings.

Dear reviewer,

We would like to sincerely thank the reviewer for their positive assessment and for the time spent evaluating our revisions. We are pleased that the added context and the clarifications regarding our methodology and findings have strengthened the manuscript. We have carefully addressed the remaining minor recommendations below to further refine the paper's clarity and nuance.

Specific suggestions and remarks

Introduction

75-76: Consider separating into two paragraphs with the species info in the next

Answer: OK, we agree and have divided the text into two paragraphs. (lines 86-87)

Methods

88-89: Be more specific as to where this study is occurring; state the country.

Answer: OK, added (line 106)

91: Expand the figure description. What is the inset map? What is it focusing in on?

Answer: OK, we have expanded the figure caption to provide better geographic context. We have clarified that the main map shows the specific study sites (colour-coded by species), while the inset map depicts the Iberian Peninsula. (lines 109-113)

122-128: Move the ethics statement up further

Answer: OK, we have relocated the Ethics Statement. It is now the first subsection within the "Materials and Methods" section (lines 98-104)

Reviewer #2: Journal: PLOS ONE

Thank you for your revision & thoughtful responses to reviewer comments. I believe that your manuscript is stronger because of these revisions. There are still some grammatical and spelling errors throughout the manuscript. Mainly this is a topic with a lot of nuances and I have listed out a couple spots below where I feel the nuance may still need to be addressed.

Dear reviewer,

We are very grateful for your positive feedback and for the recognition of the improvements made to the manuscript. We fully agree that the ecological implications of dietary studies require a high degree of nuance to avoid over-interpreting the data. Following your advice, we have carefully refined the language in the sections identified to better reflect these subtleties. Additionally, we have performed a comprehensive linguistic review to eliminate remaining grammatical and spelling errors.

Line 171: I would organise these descriptions based on the order they appear in your equation. Also your equation uses ‘pop’ and your description uses ‘pop size’ – these should be identical. I would also point out that you are using a population estimate from 2001 which is 25 years ago. Do you expect these population estimates to have changed at all? You also report that ‘other studies’ report similar population sizes between the two, but don’t cite these studies.

Answer: We have addressed each point as follows:

• We have reorganised the variable descriptions in the text to follow the exact order in which they appear in the equation. We have also unified the terminology, replacing "pop" with "pop size" in the equation to maintain consistency throughout the manuscript (lines 200-217).

• While the primary baseline for these species in the Basque Country was established by Aihartza (2001), we acknowledge the reviewer's concern regarding the age of this data. However, our research group has conducted continuous, long-term monitoring of the known colonies in the study area up to the present day. These ongoing observations suggest that while local fluctuations occur, the overall population scale has remained relatively stable over the last two decades. We have updated the text to clarify that the 2001 baseline was supplemented and validated by our recent field data (lines 211-212).

• We have added the missing citations (de Paz 2007; Migens 2007) to support the use of similar population estimates for both R. ferrumequinum and R. hipposideros. (line 215)

Line 287: I still feel a little conflicted about something that occurs in 5% of the total samples across all colonies being considered as “heavily consumed”. I understand that you have a citation to support this, but to me it still seems like it should be higher to be considered the “core diet”. Almost all of the individual pest species have a wPOO of less than 1% abundance across all sites….

Answer: We appreciate the reviewer’s critical perspective on the threshold for "core diet" and the interpretation of wPOO (weighted Percentage of Occurrence) values. However, while we agree that a 5% frequency appears low in absolute terms, this reflects the high taxonomic diversity and "long-tail" distribution typical of generalist insectivore diets captured via DNA metabarcoding. In these highly diverse dietary profiles, in insectivorous bats even the most "heavily consumed" species rarely reach high double-digit percentages because the total diet is spread across hundreds of prey taxa.

We must take into account that our diet study covers what bats ate during their active period (for many months), whereas most pest outbreaks last only a few weeks. Besides, such outbreaks are usually constrained to specific habitats or land-use types within the available foraging grounds for bats. In other words, in heterogeneous landscapes, even during outbreaks, each pest species will constitute only a fraction of prey availability for bats, for only a few weeks. Consequently, even in the most favourable consumption scenario, we shouldn't expect them to reach much higher wPOO values.

To clarify this nuance, we have revised the text. Instead of implying these pests dominate the diet in volume, we now clarify that their importance lies in their status as the most frequently and consistently detected pest taxa relative to the many other pests that appear only as "accidental" or "trace" detections (wPOO < 0.1%).

Besides, we have adjusted the wording on Line 287 (in the previous version) to replace "heavily consumed" with "consistently detected" to more accurately reflect the data. (lines 342-343 in the revised version)

Line 293: Again, “manage pest populations effectively” is a bit strong.

Answer: We agree with the reviewer that "manage pest populations effectively" implies a level of population control that was not directly measured in this study. Our results demonstrate the consumption of a wide variety of agricultural and forest pests, but do not quantify the resulting reduction in pest density. Accordingly, we have revised this sentence to reflect more accurately the provision of ecosystem services and the potential for biological suppression based on the dietary breadth of these generalist species. (line 365)

Line 324: You didn’t prove that there R.hi had a significant impact on pest species- just that they ate a lot of them. Their consumption may have been a drop in the bucket of O. sacchari abundance levels- we just don’t know.

Answer: OK, we agree that we cannot definitively state that this predation resulted in a numerical "impact" or reduction in total pest abundance. We have therefore revised the text to clarify that R. hipposideros exhibits a high frequency of predation on O. sacchari during its peak occurrence, representing a concentrated predatory pressure rather than a proven population-level impact. (line 401)

Figure 1. I think some of these site names are mixed up. Shouldn’t the labels read as Matxinbenta (MA) and Balmeseda (BA) instead of the species names? Or if that is correct, I don’t understand why and as such this should be added to the figure caption.

Answer: We agree with the reviewer and apologise for the error. We had inadvertently used species-based acronyms for these locations. We have corrected Figure 1 so that the labels now correctly reflect the site names: BA for Balmaseda and MA for Matxinbenta.

Attachment Submitted filename: RESPONSE_TO_REVIEWERS_auresp_2.docx

# Editor report: Decision Letter 2

**DOI:** 10.1371/journal.pone.0347853.r005

**Authors:** Mark Davis

8 Apr 2026

<p>Pest consumption by common bats in diverse landscapes

PONE-D-25-25129R2

Dear Dr. Aldasoro,

We’re pleased to inform you that your manuscript has been judged scientifically suitable for publication and will be formally accepted for publication once it meets all outstanding technical requirements.

Within one week, you’ll receive an e-mail detailing the required amendments. When these have been addressed, you’ll receive a formal acceptance letter and your manuscript will be scheduled for publication.

An invoice will be generated when your article is formally accepted. Please note, if your institution has a publishing partnership with PLOS and your article meets the relevant criteria, all or part of your publication costs will be covered. Please make sure your user information is up-to-date by logging into Editorial Manager at Editorial Manager® and clicking the ‘Update My Information' link at the top of the page. For questions related to billing, please contact billing support.

If your institution or institutions have a press office, please notify them about your upcoming paper to help maximize its impact. If they’ll be preparing press materials, please inform our press team as soon as possible -- no later than 48 hours after receiving the formal acceptance. Your manuscript will remain under strict press embargo until 2 pm Eastern Time on the date of publication. For more information, please contact onepress@plos.org.

Kind regards,

Mark A. Davis, Ph.D.

Academic Editor

PLOS One

Additional Editor Comments (optional):

After reviewing the Responses to Reviewrs and the revised manuscript, it's clear that the Authors have been thorough and diligent in their revisions. This manuscript now meets PLoS ONE's critera for publication, and I look forward to seeing it published.

Reviewers' comments:

# Editor report: Acceptance letter

**DOI:** 10.1371/journal.pone.0347853.r006

**Authors:** Mark Davis

PONE-D-25-25129R2

PLOS One

Dear Dr. Aldasoro,

I'm pleased to inform you that your manuscript has been deemed suitable for publication in PLOS One. Congratulations! Your manuscript is now being handed over to our production team.

At this stage, our production department will prepare your paper for publication. This includes ensuring the following:

* All references, tables, and figures are properly cited

* All relevant supporting information is included in the manuscript submission,

* There are no issues that prevent the paper from being properly typeset

You will receive further instructions from the production team, including instructions on how to review your proof when it is ready. Please keep in mind that we are working through a large volume of accepted articles, so please give us a few days to review your paper and let you know the next and final steps.

Lastly, if your institution or institutions have a press office, please let them know about your upcoming paper now to help maximize its impact. If they'll be preparing press materials, please inform our press team within the next 48 hours. Your manuscript will remain under strict press embargo until 2 pm Eastern Time on the date of publication. For more information, please contact onepress@plos.org.

You will receive an invoice from PLOS for your publication fee after your manuscript has reached the completed accept phase. If you receive an email requesting payment before acceptance or for any other service, this may be a phishing scheme. Learn how to identify phishing emails and protect your accounts at https://explore.plos.org/phishing.

If we can help with anything else, please email us at customercare@plos.org.

Thank you for submitting your work to PLOS ONE and supporting open access.

Kind regards,

PLOS ONE Editorial Office Staff

on behalf of

Dr. Mark A. Davis

Academic Editor

PLOS One
